# *StGATA14* coordinates antioxidant defense and osmotic homeostasis to enhance drought tolerance in potato

**DOI:** 10.3389/fpls.2026.1858357

**Published:** 2026-06-23

**Authors:** Xi Zhu, Xiaoqin Duan, Kaitong Wang, Yasir Majeed, Xuanrong Du, Zhilin Han, Hui Jin, Zhuo Chen, Wei Li, Shu Chen, Xidan Pang, Yu Zhang

**Affiliations:** 1 Key Laboratory of Tropical Fruit Biology, Ministry of Agriculture and Rural Affairs/Key Laboratory of Postharvest Physiology and Technology of Tropical Horticultural Products of Hainan Province, South Subtropical Crops Research Institute, Chinese Academy of Tropical Agricultural Sciences, Zhanjiang, Guangdong, China; 2 State Key Laboratory of Tropical Crop Breeding, Sanya Research Institute, Chinese Academy of Tropical Agricultural Sciences, Sanya, China; 3 State Key Laboratory of Aridland Crop Science, Gansu Agricultural University, Lanzhou, China; 4 College of Agronomy, Gansu Agricultural University, Lanzhou, China

**Keywords:** antioxidant defense, drought tolerance, photosynthetic stability, *Solanum tuberosum*, transcription factor

## Abstract

GATA transcription factors are evolutionarily conserved regulators involved in plant development and stress adaptation; however, their functional roles in potato (*Solanum tuberosum* L.) drought tolerance remain largely unclear. Here, we characterized *StGATA14*, a nucleus-localized GATA transcription factor, and investigated its role in drought stress responses. Sequence and phylogenetic analyses revealed that *StGATA14* contains a conserved GATA zinc-finger domain and clusters closely with Solanaceae homologs, suggesting functional conservation. Expression profiling revealed that *StGATA14* is significantly activated during increasing water scarcity, especially in root tissues, suggesting its role in drought-related signaling. To clarify its role, potato lines with transgene overexpression (OE) of *StGATA14* and RNA interference (RNAi) lines were generated. In drought conditions, OE plants demonstrated improved growth and biomass relative to non-transgenic (NT) controls, while RNAi lines displayed heightened sensitivity. Photosynthetic assessments showed that OE lines exhibited greater net photosynthetic rate (Pn), stomatal conductance (Gs), transpiration rate (E), and water use efficiency (WUE), reflecting enhanced photosynthetic stability. Moreover, OE plants exhibited improved antioxidant capacity, showing elevated activities of superoxide dismutase (SOD), catalase (CAT), and peroxidase (POD), alongside decreased levels of hydrogen peroxide (H_2_O_2_) and malondialdehyde (MDA). Increased levels of proline, soluble sugars, and soluble proteins indicated better osmotic regulation. At the molecular level, the overexpression of *StGATA14* substantially increased the expression of critical stress-responsive genes (*StSOD, StCAT, StPOD*, and *StP5CS*), while these genes were downregulated in RNAi lines. These findings highlight *StGATA14* as a positive regulator of drought tolerance, enhancing antioxidant defense and osmotic adjustment, and underscore its potential as a target for improving drought resilience in potatoes.

## 1 Introduction

Potato (*Solanum tuberosum* L.) is the fourth most important food crop worldwide and serves as the leading non-cereal staple crop, supporting the nutrition and livelihoods of over one billion individuals (Naz et al., 2025; Liu et al., 2026). Its significant yield capacity, short-term growth period, and remarkable nutritional benefits play a vital role in worldwide food security, especially amid rising population demands (Mir et al., 2024). However, the accelerating pace of climate change, driven by abiotic stresses, threatens to erode these advantages. Among them, drought is the most pervasive constraint on potato productivity, owing to the crop’s shallow root architecture and pronounced sensitivity of carbon metabolism to water deficit conditions (George et al., 2017). Drought triggers a cascade of physiological disruptions, including stomatal closure, photosynthetic inhibition, impaired assimilate transport, and premature canopy senescence, ultimately constraining tuber initiation and bulking (Ibrahim et al., 2024). Yield losses exceeding 50% have been reported under severe water limitation, with global production projected to decline substantially as drought frequency intensifies (Dahal et al., 2019). This challenge is exacerbated by the fact that more than half of current potato cultivation occurs in drought-prone regions, underscoring the urgency of deciphering stress-responsive regulatory networks and harnessing genetic variation from cultivated and wild germplasm (Pathak et al., 2025). Central to drought adaptation are transcriptional regulators and gene families, which integrate stress sensitivity with downstream metabolic, osmotic, and detoxification pathways to preserve cellular homeostasis and sustain yield under water-limited conditions (Parvathi and Nataraja, 2016; Keller et al., 2025).

Plant GATA transcription factors are type-IV zinc finger proteins that bind to the WGATAR cis-element through a highly conserved GATA DNA-binding domain and function as important regulators of plant growth, development, and stress adaptation (Teakle et al., 2002; Zhang et al., 2013). Increasing evidence indicates that GATA genes participate in abiotic stress responses by regulating antioxidant defense, osmotic adjustment, chlorophyll maintenance, and hormone-mediated signaling pathways, particularly abscisic acid (ABA)-dependent responses (Nutan et al., 2020; Wang et al., 2023; Yu et al., 2024). Under drought and salinity stress, the transcription of GATA genes is modulated by stress-responsive cis-regulatory elements, including ABRE and MBS motifs, enabling rapid transcriptional reprogramming and stress adaptation (Shaik and Ramakrishna, 2014; Bose et al., 2025). Functional studies in several plant species further support the role of GATA transcription factors in abiotic stress tolerance. For example, *SlGATA17* enhances salinity tolerance in tomato (Wang et al., 2023), *TaGATA62/73* improve drought and salt tolerance in wheat (Du et al., 2022), and *OsGATA16* positively regulates cold tolerance in rice (Zhang et al., 2021). These findings collectively suggest that GATA transcription factors function as important modulators of stress-responsive regulatory networks in plants.

Although genome-wide analysis has identified 57 GATA transcription factors in potato, the functional roles of most *StGATAs* in abiotic stress adaptation remain largely unknown (Zhu et al., 2024). Previous bioinformatics analysis revealed that the promoter region of *StGATA14* is enriched with multiple drought- and hormone-responsive cis-elements, including the MYB-binding drought-responsive element (MBS) and ABA-responsive elements (ABREs), suggesting its potential involvement in water-deficit signaling pathways (Zhu et al., 2024). Furthermore, preliminary qRT-PCR analysis demonstrated that *StGATA14* is among the most strongly drought-induced members of the potato GATA family. Phylogenetic analysis additionally showed that *StGATA14* shares high sequence homology with *Arabidopsis AtGATA24*, a regulator associated with stress-related photoprotection pathways (Shaikhali et al., 2012), and with sweet potato *IbGATA24*, which positively regulates drought and salt tolerance through interaction with *IbCOP9-5a* (Zhu et al., 2022). These observations collectively indicated that *StGATA14* may function as an important regulator of drought adaptation in potato. Based on these findings, the present study aimed to systematically characterize the biological function of *StGATA14* under drought stress. Our results demonstrate that *StGATA14* encodes a nuclear-localized transcription factor with conserved structural features and a distinct tissue-specific and drought-inducible expression pattern. Functional analyses further revealed that *StGATA14* positively regulates drought tolerance by enhancing antioxidant defense capacity, promoting osmotic adjustment, maintaining photosynthetic performance, and reducing oxidative damage. These physiological responses were accompanied by increased accumulation of osmoprotectants and activation of stress-responsive genes associated with reactive oxygen species (ROS) scavenging and osmotic homeostasis. Collectively, this study identifies *StGATA14* as a key positive regulator of drought adaptation in potato and provides new insights into the GATA-mediated regulatory network underlying abiotic stress tolerance in crop plants.

## 2 Materials and methods

### 2.1 Multiple-sequence alignments and phylogenetic analysis

Using CLUSTALW 1.83, a multiple-sequence alignment (MSA) of StGATA14 with orthologs from ten other plant species was performed to investigate evolutionary connections and identify conserved structural characteristics (Thompson et al., 1994). These alignments facilitated the recognition of conserved functional motifs in the examined sequences. Phylogenetic analysis was subsequently performed in MEGAX 4.1 employing the neighbor-joining approach with 1,000 bootstrap replicates to assess nodal support (Dereeper et al., 2010).

### 2.2 Plant material, growth conditions, and stress treatments

#### 2.2.1 Plant materials and culture conditions

The drought-sensitive potato (*Solanum tuberosum* L.) cultivar *‘Atlantic’* (Quandahor et al., 2021), introduced from Gansu Agricultural University (Lanzhou, Gansu, China), was used as the experimental material. Stem-node explants were established on Murashige and Skoog (MS) medium (Murashige and Skoog, 1962) enhanced with 3% (w/v) sucrose and 0.7% (w/v) agar, with the pH adjusted to 5.8–6.0. The cultures were kept in an LED illumination incubator (Model MGC-450HP, Yiheng Scientific Instrument Co., Ltd., Shanghai, China) at 22 °C under a 16−hour photoperiod (2800 lx) for three weeks. Following the excision of the apical and basal sections, 2-cm stem pieces with axillary buds were cut under sterile conditions and sub-cultured every three weeks. To induce micro-tubers, three-week-old transgenic lines (*StGATA14*-OE lines and their corresponding RNAi-knockdown lines) and NT control lines were placed on MS medium enriched with 8% sucrose and grown for six weeks. Tubers that had broken dormancy with sprout lengths around 1 mm were moved into polyethylene pots (18 cm wide, 25 cm tall) containing 3.8 kg of a 1:1 (v/v) mixture of nursery soil and vermiculite. The growth medium exhibited the following physical and chemical characteristics: pH ranged from 5.8 to 6.2, organic matter content was 13.6 g·kg^-1^, total nitrogen measured 1.4 g·kg^-1^, total phosphorus was 1.1 g·kg^-1^, total potassium amounted to 17.5 g·kg^-1^, available phosphorus stood at 60.5 mg·kg^-1^, available nitrogen was 62.5 mg·kg^-1^, and available potassium reached 165 mg·kg^-1^. Soil moisture levels were kept at 70–75% of field capacity during the establishment of the plants. All lines were grown in regulated greenhouse settings at the South Subtropical Crops Research Institute, Chinese Academy of Tropical Agricultural Sciences (Zhanjiang, Guangdong, China; 21°10′ N, 110°33′ E), under a 16-hour light (26 °C, 3400 lx)/8-hour dark (16 °C) schedule maintained by fluorescent lamps.

#### 2.2.2 Drought stress regime and experimental design

Five water-stress treatments were implemented to simulate drought stress: control (CK, 70–75% soil moisture content), mild water stress (WS1, 60–65%), moderate water stress (WS2, 50–55%), severe water stress (WS3, 40–45%), and extreme water stress (WS4, 30–35%). Soil moisture content was monitored twice daily (at 10:00 and 16:00) using a TDR−300 soil moisture sensor (Spectrum R, Aurora, IL, USA). Uniform four−week−old potted potato lines were selected for two parallel experimental series. In the first series, plants subjected to CK, WS1, WS2, WS3, and WS4 were sampled at predetermined time points (0, 1, 2, 4, 8, 16, 24, and 48 hours). Root, stem, and leaf tissues were collected at each time point, immediately frozen in liquid nitrogen, and stored at −80 °C for subsequent quantitative reverse transcription-polymerase chain reaction (qRT−PCR) analysis. In the second series, another batch of uniform four−week−old transgenic and NT potted lines were exposed to CK, WS1, WS2, WS3, and WS4 conditions for 48 hours (CK lines were maintained at 70–75% soil moisture without stress) for qRT−PCR analysis of stress-responsive genes, as well as measurements of physiological and photosynthetic parameters. Furthermore, to evaluate growth parameters of *StGATA14*-transgenic and NT lines under WS1–WS4 stress, dormancy-broken tubers of the aforementioned lines were transplanted into the same potting system and grown under CK, WS1, WS2, WS3, and WS4 conditions for four weeks (CK plants were maintained at 70–75% soil moisture without stress for 48 hours). Plant height, fresh weight, dry weight, root fresh weight, and root dry weight were statistically analyzed, respectively.

### 2.3 qRT−PCR analysis

Total RNA was extracted from frozen tissue samples using TRIzol reagent (Invitrogen, Carlsbad, CA, USA) according to the manufacturer’s instructions. Following DNase I treatment, 1 µg of total RNA was reverse-transcribed into first-strand cDNA using the First-Strand cDNA Synthesis Kit (TransGen Biotech, China). qRT-PCR was performed on a LightCycler 480-II system (Roche, Switzerland) using SYBR Premix Ex Taq (Takara, Japan). Each 20 µL reaction mixture contained 100 ng of cDNA, 0.8 µM of each gene-specific primer, and 10 µL of SYBR Premix. The amplification protocol consisted of an initial denaturation at 94 °C for 2 min, followed by 34 cycles of 94 °C for 30 s, 60 °C for 34 s, and 72 °C for 30 s, with fluorescence data acquired at each extension step. Transcript levels were normalized to the potato elongation factor 1-alpha (*StEF1α*) gene as an internal control. Relative expression was calculated using the 2^−ΔΔCt^ method (Livak and Schmittgen, 2001). For each experimental condition, nine biological replicates, each with three technical replicates, were assessed. All primer sequences used for qRT-PCR are listed in **Supplementary Table 1**.

### 2.4 Subcellular localization of StGATA14 in *Nicotiana benthamiana* epidermal cells

The subcellular localization of StGATA14 was assessed by amplifying its entire coding sequence using gene-specific primers (**Supplementary Table 1**) and ligating the amplicon into the pCAM35s-GFP vector. This created a translational fusion between StGATA14 and green fluorescent protein (GFP), with expression driven by the cauliflower mosaic virus 35S promoter. The resulting recombinant plasmid (pCAM35s-StGATA14-GFP) was verified by sequencing and subsequently introduced into *Agrobacterium tumefaciens* strain GV3101 via electroporation. For transient expression assays, *Agrobacterium* cultures harboring the StGATA14-GFP fusion construct were infiltrated into fully expanded leaves of four-week-old *Nicotiana benthamiana* plants following established protocols (Sparkes et al., 2006). Infiltrated plants were maintained under standard growth conditions for 48 h before visualization. GFP fluorescence was examined using a Leica TCS SP8 confocal laser scanning microscope (Leica Microsystems, Wetzlar, Germany) with excitation at 488 nm and emission detected at 500–530 nm. Images were taken and analyzed with a Leica TCS SP8 confocal laser scanning microscope (Leica Microsystems, Germany). The primers utilized for constructing the vector are listed in **Supplementary Table 1**.

### 2.5 Construction of expression vectors and genetic transformation of potato

#### 2.5.1 Vector construction for *StGATA14* overexpression and RNAi silencing

To produce transgenic potato lines with altered *StGATA14* expression, constructs for both *StGATA14*-OE and RNAi were created. The complete coding sequence of *StGATA14* (GenBank accession: XM_006346941.2) was augmented via PCR with specific primers (**Supplementary Table 1**; synthesized by Bioeditas, Shaanxi, China) and subsequently cloned into the pBI121-EGFP binary vector according to recognized procedures (Li et al., 2020). For the assembly of RNAi constructs, an RNAi vector specific to the fragment was created based on previously outlined methods (Lu et al., 2019). The sense fragment of *StGATA14*, amplified using primers containing *Bam* HI and *Kpn* I restriction sites, was inserted into the pHANNIBAL intermediate vector to create pHAN-StGATA14-R. The antisense fragment, amplified with primers featuring *Hind* III and *Xba* I sites, was then inserted into the same vector to create pHAN-StGATA14-RF. The entire RNAi expression cassette containing both sense and antisense sequences was cut out and sub-cloned into the pART vector with *Bam* HI and *Xba* I restriction enzymes, resulting in the final construct pART-StGATA14-RNAi. The restriction maps for the pKANNIBAL RNAi intermediate vector and the pART binary plant expression vector are presented in​ **Supplementary Figures 1**, **2**, respectively.

#### 2.5.2 *Agrobacterium*-mediated transformation

*Agrobacterium tumefaciens* strain LBA4404 was exposed to the resultant recombinant plasmids (pBI121-StGATA14-EGFP and pART-StGATA14-RNAi). Transformed bacterial cells were selected on LB agar supplemented with 50 mg L^-1^ spectinomycin and 50 mg L^-1^ gentamicin following 48 h incubation at 28 °C. Positive transformants were verified by colony PCR using construct-specific primer pairs (**Supplementary Table 1**). For plant transformation, bacterial cultures were grown to logarithmic phase in LB medium containing appropriate antibiotics, harvested by centrifugation at 5,000 × g for 10 min, and resuspended in liquid MS medium to a final optical density (OD_600_) of 0.3.

#### 2.5.3 Potato transformation and regeneration

**Figure 1:**
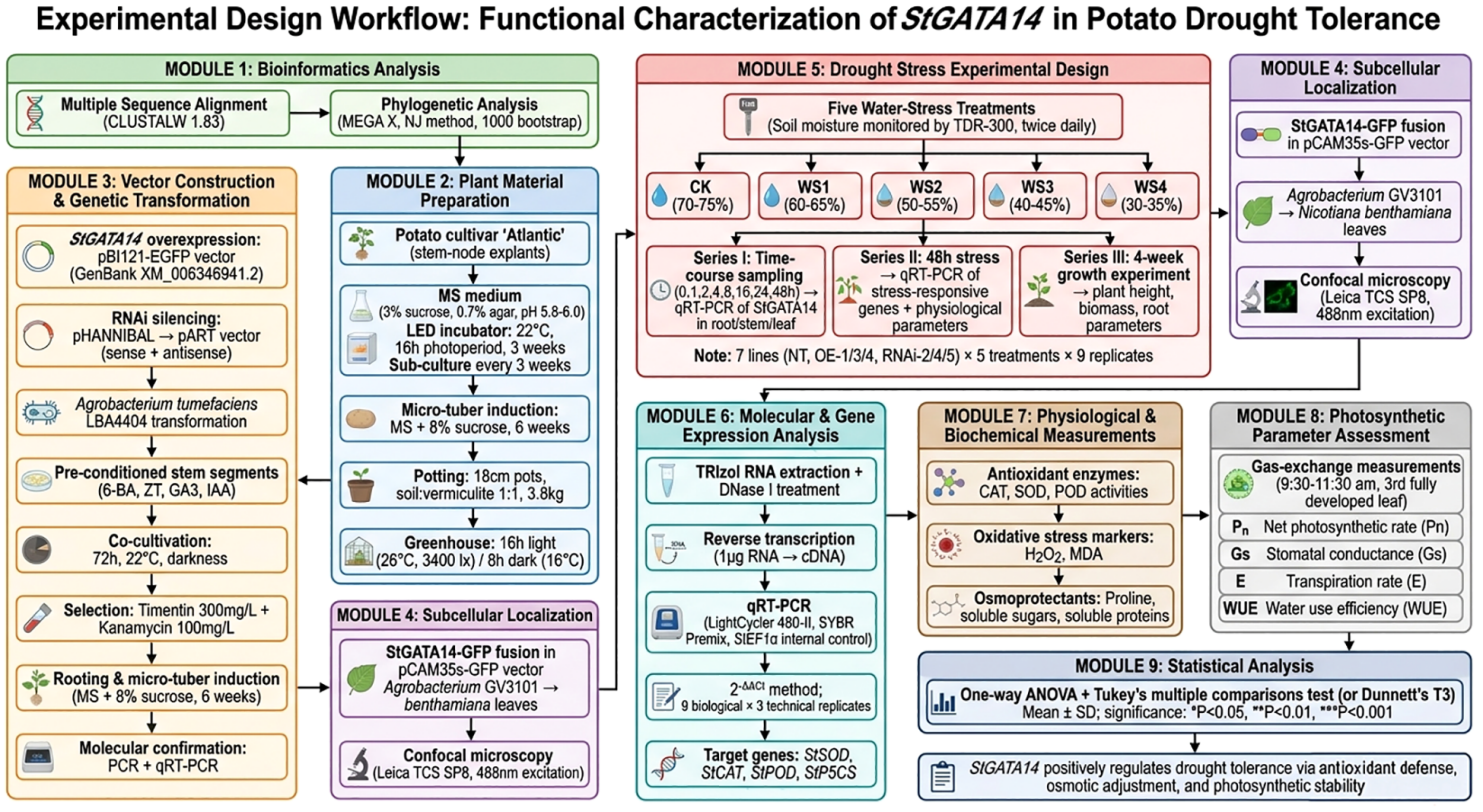
Schematic of the experimental workflow for validating the drought-resistance function of *StGATA14* in transgenic potato plants.

Surface-sterilized potato stem segments (approximately 2 cm in length) derived from the cultivar ‘*Atlantic*’ were pre-conditioned on solid MS medium supplemented with 30 g L^-1^ sucrose, 7.4 g L^-1^ agar, 0.5 mg L^-1^ 6-benzylaminopurine (6-BA), 2 mg L^-1^ zeatin (ZT), 0.2 mg L^-1^ gibberellic acid (GA_3_), and 1 mg L^-1^ indole-3-acetic acid (IAA) at pH 5.8. Explants were immersed in the *Agrobacterium* suspension for 10–15 min, blotted dry on sterile filter paper, and co-cultivated on the same medium for 72 h in darkness at 22 °C. Following co-cultivation, explants were transferred to selection medium comprising MS basal salts, 30 g L^-1^ sucrose, 7.4 g L^-1^ agar, 0.5 mg L^-1^ 6-BA, 2 mg L^-1^ ZT, 0.2 mg L^-1^ GA_3_, 1 mg L^-1^ IAA, 300 mg L^-1^ Timentin, and 100 mg L^-1^ kanamycin (pH 5.8). Sub-culturing onto fresh selection medium was performed at two-week intervals. Regenerated kanamycin-resistant shoots (approximately 2–3 cm in height) were excised and transferred to rooting medium containing MS salts, 30 g L^-1^ sucrose, 7.4 g L^-1^ agar, 300 mg L^-1^ Timentin, and 100 mg L^-1^ kanamycin (pH 5.8). The detailed experimental protocol for the genetic transformation of potato plants is provided in **Supplementary Figure 3**. The molecularly confirmed positive transgenic plantlets were used to obtain uniform experimental microtubers via the *in vitro* microtubers induction technique (MS + 8% sucrose, cultured for 6 weeks). Following dormancy break (sprout length ~1 mm), these microtubers were used for subsequent pot-based stress experiments. A schematic diagram detailing the experimental design for validating the drought resistance function of *StGATA14* in transgenic potato plants is shown in **Figure 1**.

#### 2.5.4 Molecular confirmation of transgenic lines

To validate the integration of the *StGATA14*-transgene in candidate transgenic potato lines, PCR analysis was performed. For *StGATA14*-OE lines, the specific primer pair OE-F (5’-ATCTTCGTCAACATGGTGGA-3’) and OE-R (5’-ACTTCGAGAGCTTTTTCCGGTA-3’) was used to amplify a fragment spanning the endogenous *StGATA14*. For RNAi-knockdown lines, the primer pair RNAi-F (5’-GATTACTCCGGCAACTGTCAA-3’) and RNAi-R (5’-TGAGATAACCATTCTAGGTTAT-3’) was employed to confirm the successful incorporation of the RNAi cassette. Following positive PCR confirmation, qRT-PCR was directed to determine the transcriptional expression level of *StGATA14* in each candidate transgenic line, using NT lines as the control baseline. This allowed systematic evaluation of the gene overexpression efficiency or knockdown efficiency in each line. Stable transgenic lines exhibiting the expected expression patterns were selected and subsequently maintained through periodic subculture on MS medium under controlled growth chamber conditions. This *in vitro* propagation and long-term preservation ensured a consistent genetic background and phenotypically stable experimental material for the succeeding role of *StGATA14*.

### 2.6 Evaluation of growth parameters under drought stress conditions

To evaluate the effects of water deficit on the growth traits of potato, this study conducted a systematic quantitative analysis of growth parameters on the *StGATA14*-transgenic and NT lines of the *‘Atlantic’* cultivar, which were cultivated under controlled greenhouse conditions as described previously. All measurements were conducted with appropriate biological replication to ensure statistical robustness (A completely randomized design was used for this experiment, which was comprised seven potato lines and five treatment groups, with each treatment replicated nine times. Each replicate consisted of three pots, with one plant grown per pot). Plant height was determined by measuring the vertical distance from the soil surface to the apical meristem of the main stem using a calibrated metric scale. Upon harvest, whole plants were gently removed from pots, and root systems were carefully washed to remove adherent substrate particles. Fresh weights of both entire plants and excised root systems were recorded immediately using an analytical balance (precision ±0.001 g). Dry mass determination was performed following established protocols (Qiu et al., 2023), with minor modifications. Plant tissue samples were initially subjected to 105 °C for 10 min to rapidly inactivate endogenous enzymatic activities and terminate respiratory metabolism. Subsequently, samples were transferred to a forced-air drying oven maintained at 80 °C until constant weight was achieved (typically 48–72 h). Dry weights were recorded after cooling to ambient temperature in a desiccator to prevent moisture absorption. This approach ensured accurate determination of biomass accumulation patterns in response to differential water availability across experimental treatments.

### 2.7 Assessment of physiological and biochemical parameters under drought stress

Four-week-old potted transgenic and NT potato lines were exposed to diverse drought stress treatments (WS1–WS4) for 48 hours, with CK serving as the control. The fifth to sixth fully expanded functional leaves beneath the shoot apex were harvested for physiological measurements. Measurements of CAT, SOD, and POD activities were carried out according to the procedures established by Li (2000). The quantification of H_2_O_2_, proline, and MDA was performed using the methods reported by Loreto and Velikova (2001); Bates et al. (1973), and Heath and Packer (1968), respectively. Soluble sugar and soluble protein contents were evaluated following the procedures of DuBois et al. (1956) and Bradford (1976), respectively. For full methodological details, see **Supplementary Data 1**.

### 2.8 Analysis of photosynthetic parameters under drought stress

To assess the effect of water scarcity on photosynthetic efficiency, gas-exchange metrics were recorded in *StGATA14*-transgenic and NT ‘*Atlantic*’ potato cultivar cultivated under the drought treatments and growth conditions described earlier. Measurements took place from 9:30 to 11:30 a.m., and the third fully developed leaf from the top of each plant was chosen for examination. Leaf gas-exchange measurements were taken with a LI-6400XT portable photosynthesis system (Li-COR Inc., Lincoln, NE, USA). Essential physiological parameters such as Pn, E, Gs, and WUE were assessed. The experimental chamber was maintained with a photosynthetic photon flux density (PPFD) of 1500 μmol m^-2^ s^-1^, a CO_2_ concentration of 400 μmol mol^-1^, relative humidity ranging from 60% to 70%, and an air flow rate of 500 μmol s^-1^ throughout the measurement period.

### 2.9 Statistical analysis

The GraphPad Prism version 10.6.1 (GraphPad Software, Boston, MA, USA) and IBM SPSS Statistics 19.0 (IBM Corporation, Chicago, IL, USA) were used for statistical analysis and data processing. Nine biological replicates were used per experiment, with results expressed as mean ± standard deviation (SD). Two-way analysis of variance (ANOVA) was used to compare differences across treatments and genotypes. Tukey’s *post hoc* test was applied for multiple-group comparisons, when significant main effects were identified. Significance levels were defined as *P < 0.05, **P < 0.01, and ***P < 0.001. Illustrations were finalized and formatted for publication using Adobe Illustrator 2022.

## 3 Results

### 3.1 Multiple sequence alignment and phylogenetic analysis

**Figure 2:**
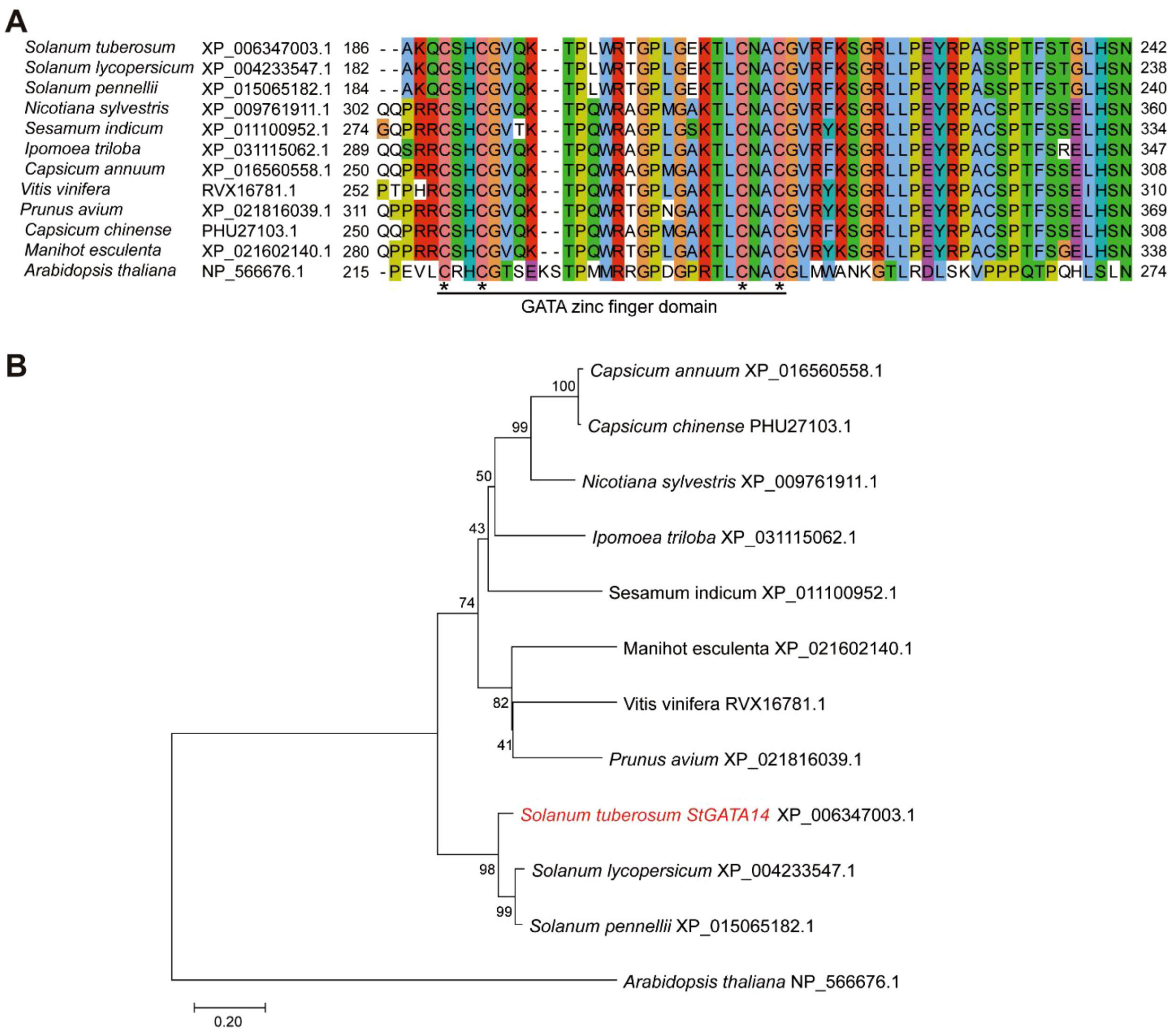
Sequence alignment and phylogenetic analysis of StGATA14 proteins from different plant species. **(A)** Multiple sequence alignment of StGATA14 and its homologous proteins from several plant species, including *Solanum tuberosum, Solanum lycopersicum, Solanum pennellii, Nicotiana sylvestris, Capsicum annuum, Capsicum chinense, Ipomoea triloba, Sesamum indicum, Manihot esculenta, Vitis vinifera, Prunus avium*, and *Arabidopsis thaliana*. The conserved GATA zinc finger domain is highlighted, showing the characteristic cysteine residues responsible for zinc ion coordination and DNA-binding activity. The high level of conservation across species indicates the functional importance of this domain. **(B)** Phylogenetic tree construction showing the evolutionary relationships among StGATA14 proteins from different plant species. The tree was constructed based on full-length protein sequences, and bootstrap values are indicated at each node. *Solanum tuberosum* StGATA14 clusters closely with its homologs from *Solanum lycopersicum* and *Solanum pennellii*, suggesting strong evolutionary conservation within the Solanaceae family. The unrooted tree was constructed using the neighbor-joining method in MEGA 4.1. Bootstrap values exceeding 50% from 1000 replicates are shown at each branch.

Multiple sequence alignment revealed that the StGATA14 protein from *Solanum tuberosum* shares a highly conserved GATA zinc finger domain with its orthologs from other plant species. This conserved region contains the characteristic cysteine-rich motif responsible for zinc ion coordination and DNA binding. The alignment shows that the core residues within this motif are strongly conserved across all examined species, including *Solanum lycopersicum, Solanum pennellii, Capsicum annuum, Capsicum chinense, Nicotiana sylvestris, Ipomoea triloba, Sesamum indicum, Manihot esculenta, Vitis vinifera, Prunus avium*, and *Arabidopsis thaliana* (**Figure 2A**). The protein IDs of these species, along with potato, are listed in **Supplementary Table 2**. The high degree of conservation in this domain suggests that these proteins likely retain similar DNA-binding functions and regulatory roles. Although minor variations are present in the surrounding amino acid sequences, the integrity of the zinc finger motif remains unchanged, indicating strong evolutionary pressure to maintain this functional region. Phylogenetic analysis further clarifies the evolutionary relationships among these proteins. The phylogenetic tree groups the proteins into several clades according to their sequence similarity. Notably, *Solanum tuberosum* StGATA14 clusters closely with its homologs from *Solanum lycopersicum* and *Solanum pennellii* (**Figure 2B**), with high bootstrap support, indicating a strong evolutionary relationship among members of the Solanaceae. This clustering suggests that these species share a recent common ancestor for this gene and may possess similar functional characteristics. In contrast, proteins from other species, such as *Capsicum*, *Nicotiana*, and *Ipomoea*, form nearby but distinct branches, while more distantly related species, including *Manihot esculenta*, *Vitis vinifera*, and *Prunus avium*, are positioned in separate clades. *Arabidopsis thaliana* appears as a more distant outgroup in the tree, reflecting its greater evolutionary divergence from the Solanaceae lineage. Overall, the strong conservation of the GATA zinc finger domain combined with the phylogenetic clustering of closely related species indicates that StGATA14 is evolutionarily conserved and may perform similar regulatory functions across different plant species, particularly within the Solanaceae family.

### 3.2 Expression patterns of *StGATA14* in various tissues of potato under different drought stress conditions

**Figure 3:**
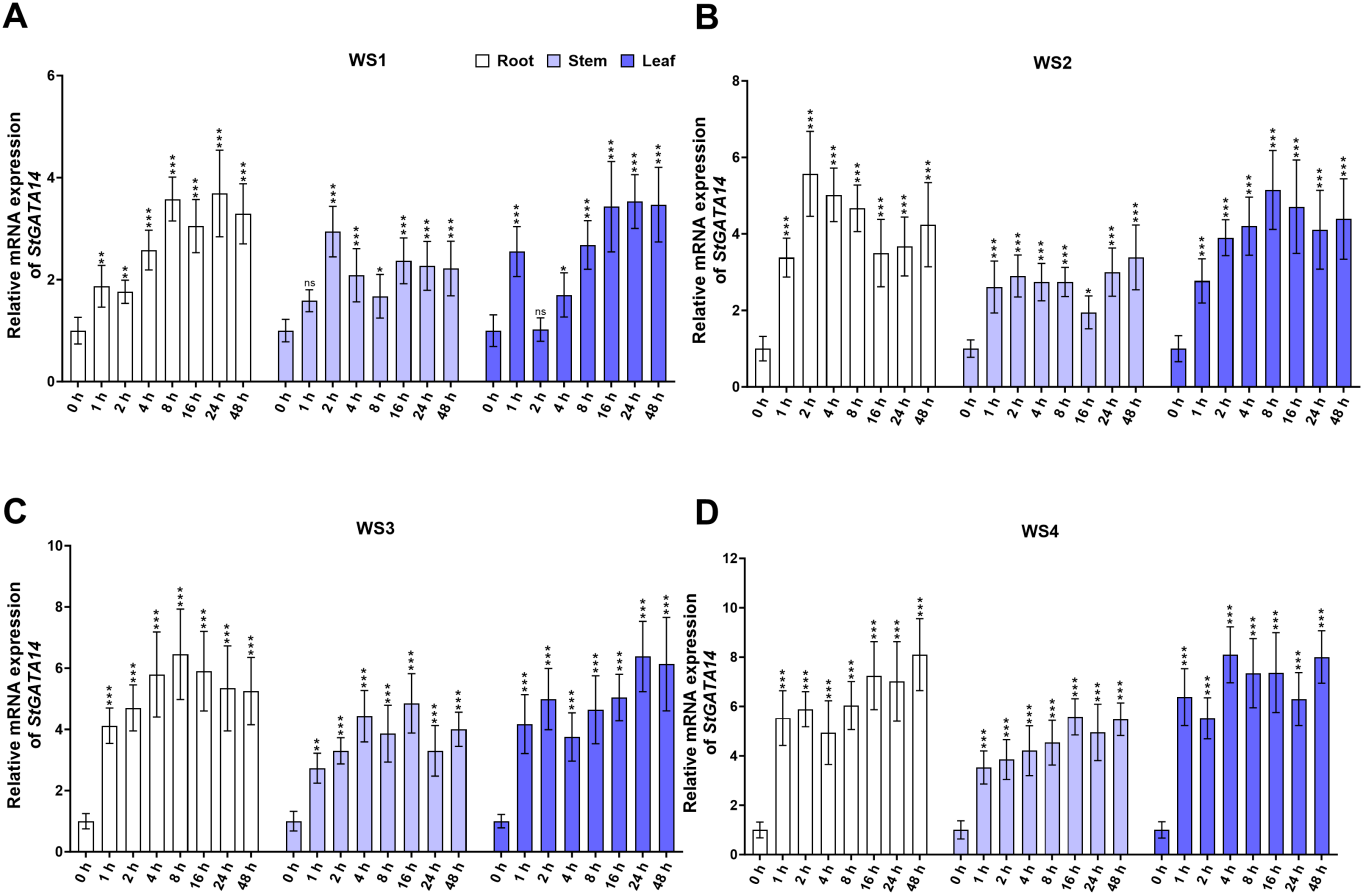
Tissue-specific expression profiles of *SGTA14* under four experimental conditions [WS1; 60–65% (CK), WS2; 50–55%, WS3; 40–45%, and WS4; 30–35%]. Relative mRNA expression levels of the *SGTA14* gene were measured in root, stem, and leaf tissues at 0 h, 1 h, 2 h, 4 h, 8 h, 16 h, 24 h, and 48 h. Plants were subjected to four distinct treatment conditions: **(A)** WS1; CK, **(B)** WS2, **(C)** WS3, and **(D)** WS4. Data are presented as mean ± standard deviation (n = 9); different letters indicate significant difference (ns, non-significant; **P < 0.01, ***P < 0.001, by one-way ANOVA with Tukey test or Dunnett’s T3 for *post hoc* analysis) among various tissues. Tissue samples were collected at the indicated time points (hours post-treatment), and *SGTA14* transcript abundance was quantified using qRT-PCR.

To elucidate the transcriptional expression profile of *StGATA14* under drought stress, this study implemented a gradual drought stress treatment with four soil moisture gradients (WS1 to WS4) on potato plants. The temporal expression patterns of this gene in root, stem, and leaf tissues were systematically analyzed at multiple time points (0, 1, 2, 4, 8, 16, 24, 48 h) to clarify its transcriptional regulatory characteristics in drought response. Under mild drought stress (WS1, **Figure 3A**), the expression of *StGATA14* in roots began to rise progressively 4 hours after stress initiation, reaching a significant peak around 24 h, followed by a moderate decline while maintaining a level above the baseline. In stems, expression showed a continuous, low-amplitude, and steady upregulation throughout the 48-h period, with only a slight increase at 2 h. Leaves exhibited a similar overall trend to stems, displaying relatively stable upregulation at 16, 24, and 48 h. Under moderate drought (WS2, **Figure 3B**), the overall expression trend resembled that of WS1: in roots, transcriptional activation occurred earlier, beginning around 2 h; expression declined at intermediate time points such as 16 and 24 h, followed by a secondary upsurge at 48 h. In stems, expression was generally upregulated throughout, peaking at 48 h with only a slight decrease at 16 h. In leaves, significant upregulation was observed from the early stress stage, with a distinct expression peak at 8 h. Under severe drought (WS3, **Figure 3C**), the expression dynamics of *StGATA14* became more pronounced. In roots, induction occurred more rapidly, with significant upregulation observed at 2 and 4 h, and expression peaking at 8 h; levels declined slightly from 16 to 48 h but remained significantly higher than the initial level at 0 h. Stem expression showed a synchronous transient increase with early root activation, peaking at 4 h, followed by another marked upregulation at 16 h. Leaves exhibited upregulation throughout the treatment period, with a significant peak at 24 h. Under extreme drought (WS4, **Figure 3D**), the transcriptional response of *StGATA14* was most intense. Roots displayed strong and sustained upregulation, with expression rising sharply after 8 h and remaining at very high levels until 48 h. Stem expression increased continuously from the early stress stage until 24 h, peaking at 16 h. Leaves also showed clear induction: expression began to rise at 1 h, dipped briefly at 2 h, then increased steadily from 4 to 48 h, with only a minor fluctuation at 24 h. Drought stress significantly induced the expression of *StGATA14* in potato root, stem, and leaf tissues, indicating that this gene may play a crucial role in drought stress response and tolerance regulation in potato.

### 3.3 Subcellular localization of StGATA14 and construction of transgenic (overexpression and RNA-interference) plant of potato

**Figure 4:**
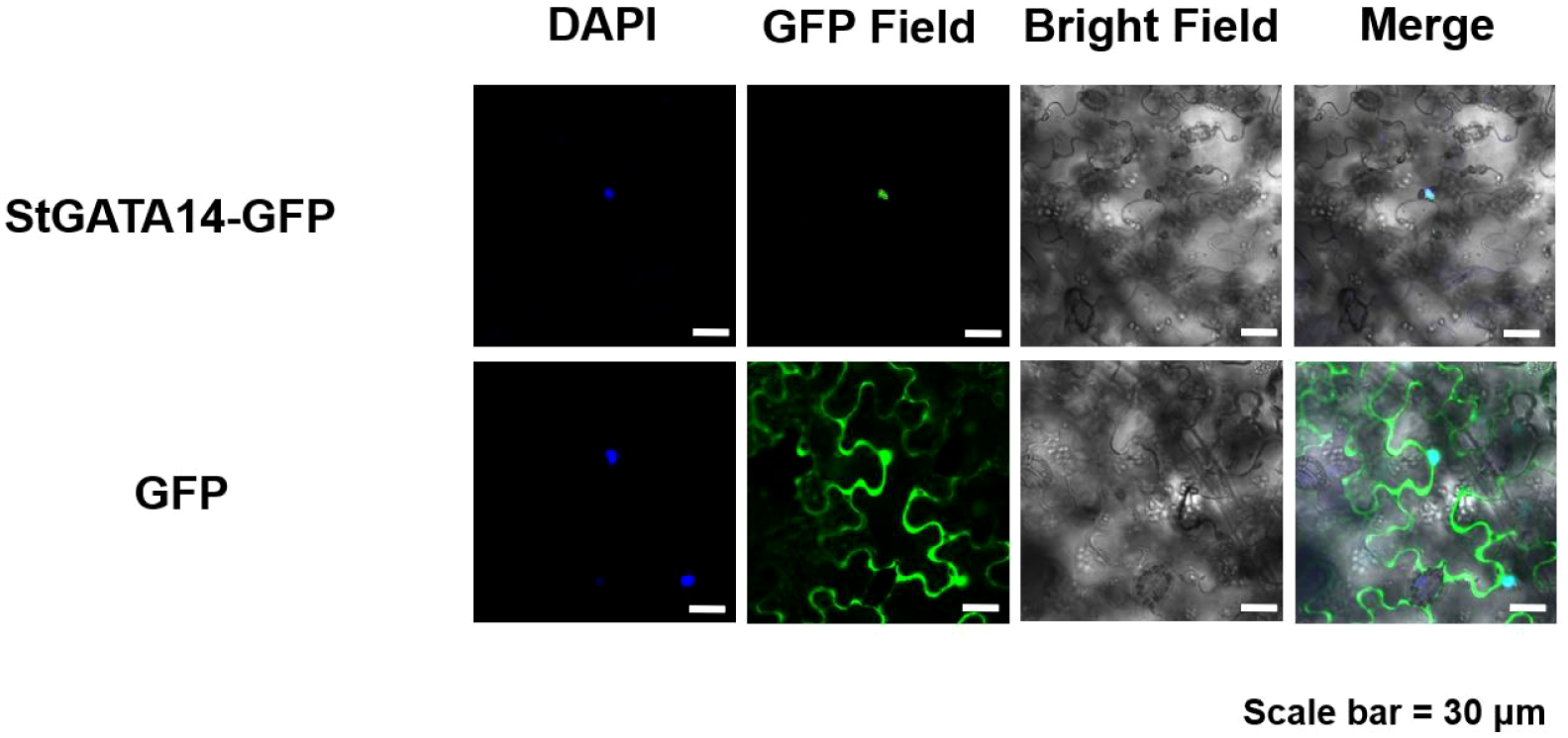
StGATA14 localizes to the nucleus. Subcellular distribution of the StGATA14-GFP fusion protein in *Nicotiana benthamiana* leaf epidermal cells. The StGATA14 coding sequence was fused in-frame with GFP and transiently expressed under the control of the CaMV-35S promoter. Free GFP served as a control. Confocal imaging revealed that while free GFP signal was diffusely distributed throughout the cytoplasm and nucleus, the StGATA14-GFP fusion protein accumulated exclusively within the nucleus. Scale bar = 30 μm.

To investigate the subcellular localization of StGATA14, the complete coding sequence was translationally fused to GFP and placed under the regulation of the CaMV 35S promoter. This StGATA14–GFP fusion construct, together with a free GFP control, was introduced into *Nicotiana benthamiana* leaf epidermal cells via *Agrobacterium tumefaciens* (strain GV3101)-mediated transient transformation. Fluorescence signals were examined using confocal laser scanning microscopy 48 h after infiltration. As expected, the control GFP protein exhibited a diffuse distribution throughout the cell membrane, cytoplasm, and nucleus (**Figure 4**). In contrast, after DAPI staining for specific nuclear labeling, subcellular localization analysis of the transformed cells revealed that the fluorescence signal of the StGATA14-GFP fusion protein was exclusively localized to the nucleus, with no significant fluorescence signal detected in other cellular compartments such as the cell membrane or cytoplasm. This nuclear-restricted localization is consistent with the predicted function of StGATA14 as a transcriptional regulator and supports its potential role in modulating gene expression during drought stress responses in potato.

For functional characterization of *StGATA14* in stress adaptation, transgenic potato lines of cultivar ‘*Atlantic*’ were produced, and both OE and RNAi-knockdown constructs targeting *StGATA14* were generated to evaluate the phenotypic effects of enhanced and reduced expression levels. The reverse transcription–polymerase chain reaction (RT-PCR) was utilized to validate the transgene in the potato genome (**Supplementary Figures 4A, C**). Subsequent qRT-PCR analysis revealed substantial alterations in the transcript abundance of *StGATA14* in the transgenic lines. Specifically, the expression of *StGATA14* in the OE lines increased by approximately 14.6- to 15.9-fold relative to the NT control lines (***P < 0.001; **Supplementary Figure 4B**). On the contrary, a marked declined in the transcript level was exhibited in the RNAi lines, with expression suppressed by approximately 66%–72% compared to the NT lines (***P < 0.001; **Supplementary Figure 4D**). Based on these expression profiles, three OE lines (OE-1, OE-3, and OE-4) exhibiting the highest transcript accumulation and three RNAi lines (RNAi-2, RNAi-4, and RNAi-5) displaying the strongest suppression of expression were selected for subsequent functional analyses under drought stress conditions. These lines provided a suitable experimental system for further elucidating the role of *StGATA14* in drought stress tolerance in potato.

### 3.4 *StGATA14* modifies drought tolerance by maintaining the growth and biomass of potatoes

**Figure 5:**
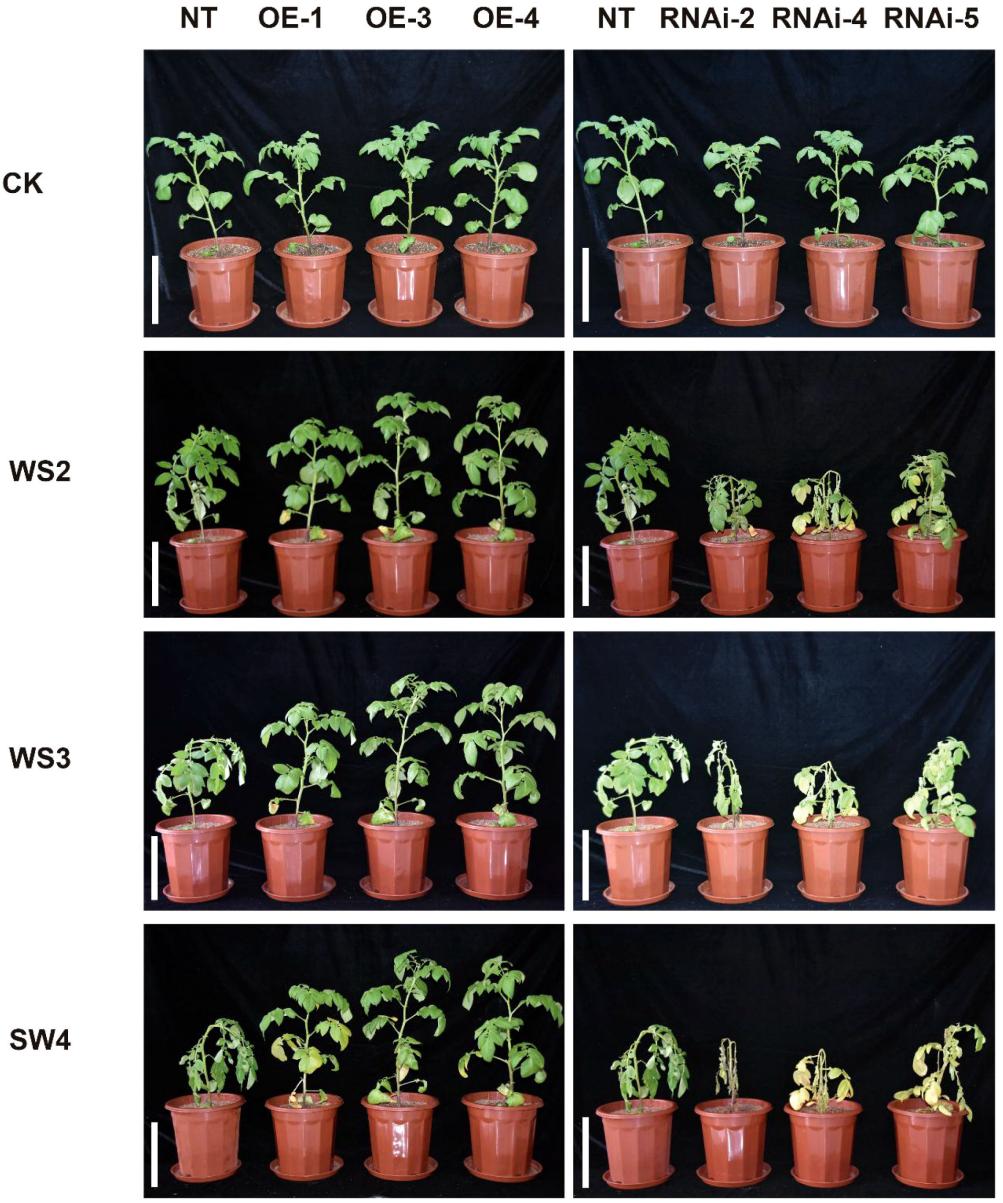
Phenotypic comparison of potato transgenic and non-transgenic lines under different water stress condition. The panel includes control (CK); 70–75%, water stress 1; 60–65%, water stress 2; 50–55%, water stress 3; 40–45%, water stress 4; 30–35% soil moisture, *StGATA14* overexpression lines (OE-1, OE-3, OE-4), and RNAi-silenced lines (RNAi-2, RNAi-4, RNAi-5).

To investigate the contribution of *StGATA14* to drought tolerance, *in vitro* plantlets of the potato cultivar ‘*Atlantic*’ were used, including NT lines, *StGATA14*-OE lines (OE-1/3/5), and RNAi lines (RNAi-2/4/5). These plants were grown for four weeks under progressively increasing water-deficit conditions (WS1–WS4), and multiple growth-related traits, including plant height, fresh and dry weights of plants and their roots, were evaluated to assess phenotypic responses. Under control conditions (CK; 70–75% soil moisture), NT, OE, and RNAi lines did not reveal any significant results for any of the measured growth parameters (**Supplementary Table 3**), indicating that altered expression of *StGATA14* did not affect normal plant development under optimal water availability. However, clear differences emerged when plants were exposed to moderate water stress (WS1; 60–65% soil moisture). Under this condition, most growth parameters, including biomass accumulation and overall plant vigor, were significantly higher in the OE lines compared with NT and RNAi lines (***P < 0.001, **P < 0.01, *P < 0.05). In opposition, marked reductions in growth traits was observed in RNAi lines, and clear symptoms of stress-induced growth inhibition were shown (**Supplementary Table 3**). Although plant height remained largely unchanged among most genotypes, the RNAi-2 line showed a modest but significant reduction compared with NT lines (*P < 0.05). Fresh weight measurements further supported these trends, with OE lines maintaining higher biomass under stress, although the OE-3 line showed no significant difference relative to NT lines. When water availability was further reduced (WS2; 50–55% soil moisture), all genotypes exhibited some degree of growth suppression, reflecting the increasing severity of drought conditions. However, in comparison to NT lines, the *StGATA14*-OE lines continuously maintained much greater growth and biomass (***P < 0.001, **P < 0.01, *P < 0.05). RNAi lines, on the other hand, showed a significant decrease in these parameters, indicating a higher sensitivity to water constraint (**Supplementary Table 3**; **Figure 5**). Under more severe water deficit (WS3; 40–50% soil moisture), visible wilting symptoms became evident in all lines, indicating disruption of water balance. Despite these conditions, OE lines continued to display relatively improved growth performance and less severe phenotypic damage compared with NT lines (**Supplementary Table 3**; **Figure 5**), while RNAi lines exhibited stronger growth inhibition and more pronounced wilting symptoms. At the highest level of stress (WS4; 30-35% soil moisture), drought symptoms intensified considerably. NT and RNAi lines showed severe physiological damage, including leaf yellowing and tissue wilting progressing to necrosis (**Figure 5**), whereas OE lines retained comparatively better structural integrity and maintained higher values for several growth-related parameters (**Supplementary Table 3**). These findings indicate that *StGATA14* positively influences plant growth and biomass maintenance under water-limited conditions. The contrasting phenotypes observed between OE and RNAi lines strongly support a functional role for *StGATA14* in regulating drought stress tolerance in potato.

### 3.5 *StGATA14* modulates drought tolerance by retaining physiological and biochemical responses of potato to drought stress

**Figure 6:**
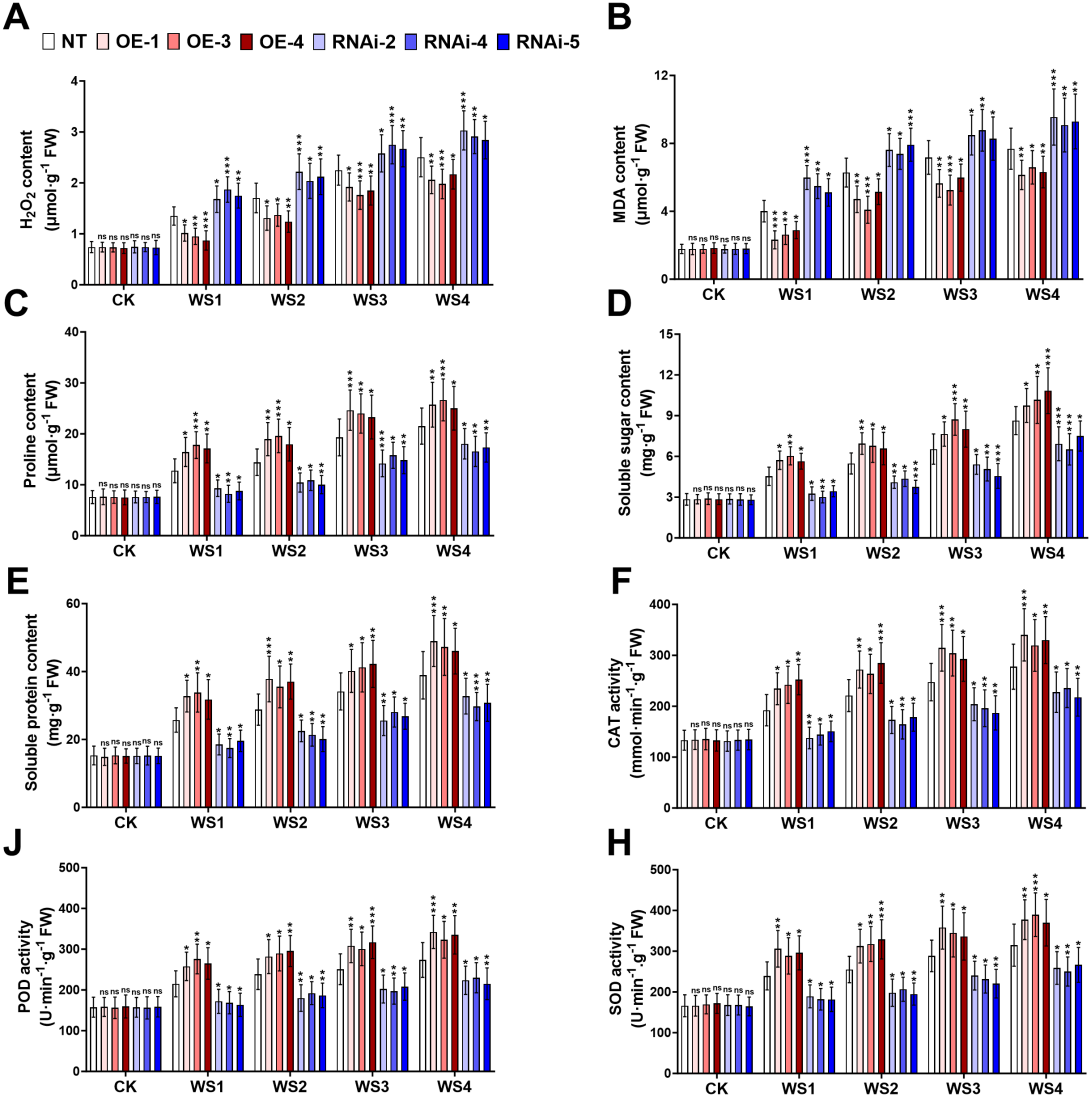
*StGATA14* modifies potato physiological indicators of cultivar *Atlantic*; **(A)** H_2_O_2_ content, **(B)** MDA content, **(C)** proline content, **(D)** soluble sugar content, **(E)** soluble protein content **(F)** CAT, **(G)**, POD, and **(H)** SOD activities after exposure to drought stress treatments (CK: 70–75%; WS1: 60–65%; WS2: 50–55%; WS3: 40–45%; WS4: 30–35%). NT, non-transgenic plants; OE, pBI121-EGFP-StGATA14-transgenic plants (OE-1, OE-3, and OE-5); RNAi, pART-StGATA14-RNAi-transgenic plants (RNAi-2, RNAi-4 and RNAi-5). The data are presented as mean ± standard deviation. Data are presented as mean ± standard deviation (n = 9). Statistical significance was determined by one-way ANOVA followed by Tukey’s multiple comparisons test, with “ns” indicating no significant difference, *P < 0.05, **P < 0.01, ***P < 0.001.

To elucidate the contribution of *StGATA14* to drought tolerance, physiological and biochemical responses were examined in *StGATA14-*OE, RNAi-knockdown, and NT potato lines. Several key indicators associated with drought stress were evaluated, including antioxidant enzyme activities (SOD, POD, CAT), oxidative stress markers (H_2_O_2_, MDA), and osmoprotectant (proline, soluble sugars, soluble proteins). These parameters were measured under control conditions and under progressively increasing drought stress levels (WS1-WS4). Under optimal growth conditions (CK; 70-75% soil moisture), no significant differences were detected among OE, RNAi, and NT lines for any of the measured physiological or biochemical traits (**Figures 6A–H**). This observation suggests that altered expression of *StGATA14* does not interfere with normal cellular metabolism or basal physiological status in the absence of stress.

When plants were subjected to mild drought stress (WS1; 60–65% soil moisture), clear differences among genotypes began to emerge. Although all plants initiated stress-related adjustments, OE lines (OE-1/3/5) accumulated significantly lower H_2_O_2_ content (**Figure 6A**) and MDA content (**Figure 6B**) than NT lines, indicating reduced oxidative damage and improved membrane stability. In parallel, OE lines exhibited higher accumulation of osmoprotective compounds, including proline (**Figure 6C**), soluble sugars (**Figure 6D**), and soluble proteins (**Figure 6E**). The activities of antioxidant enzymes (CAT, POD, and SOD; **Figures 6F–H**) were also markedly higher in OE lines. In contrast, RNAi lines (RNAi-2/4/5) displayed increased oxidative stress markers and reduced levels of osmolytes and antioxidant enzyme activities, indicating a weaker stress response. As drought stress progressed to moderate levels (WS2; 50-55% soil moisture), the physiological differences among genotypes became more pronounced. Although oxidative stress increased in all plants, OE lines consistently maintained lower H_2_O_2_ (**Figure 6A**) and MDA (**Figure 6B**) accumulation compared with NT lines. At the same time, the levels of proline (**Figure 6C**), soluble sugars (**Figure 6D**), and soluble proteins (**Figure 6E**) remained significantly higher in OE lines, suggesting improved osmotic regulation under water deficit conditions. Correspondingly, antioxidant enzyme activities remained strongly elevated in OE lines (**Figures 6F–H**), whereas RNAi lines exhibited substantially reduced enzymatic activity and greater oxidative damage. Under severe drought stress (WS3; 40–45% soil moisture), the beneficial effects of *StGATA14*-OE were even more evident. OE lines continued to maintain lower oxidative stress levels (**Figures 6A, B**) and higher osmolyte accumulation (**Figures 6C–E**) relative to NT lines. Enhanced antioxidant enzyme activity was also sustained in OE lines (**Figures 6F–H**), indicating an efficient ROS detoxification system. In contrast, NT lines showed clear signs of stress-induced physiological imbalance, while RNAi lines exhibited the highest levels of oxidative damage and the weakest protective responses. At the most extreme drought condition (WS4; 30–35% soil moisture), the contrasting phenotypes among the genotypes became particularly clear. OE lines retained relatively lower levels of oxidative damage (**Figures 6A, B**) and maintained higher concentrations of osmoprotectants (**Figures 6C–E**) and antioxidant enzyme activities (**Figures 6F–H**) compared with NT lines. These responses likely contributed to the preservation of cellular integrity under severe dehydration. Conversely, RNAi lines showed the strongest stress symptoms, characterized by excessive ROS accumulation and a markedly reduced antioxidant and osmotic defense capacity. Overall, the physiological and biochemical profiles observed across the drought gradient indicate that *StGATA14* positively regulates drought tolerance in potato. Overexpression of this gene enhances osmotic adjustment and antioxidant defense, thereby limiting oxidative damage under water deficit conditions, whereas suppression of *StGATA14* compromises these protective mechanisms and increases drought sensitivity.

### 3.6 *StGATA14* confers drought tolerance through transcriptional regulation of stress-responsive genes in potato

**Figure 7:**
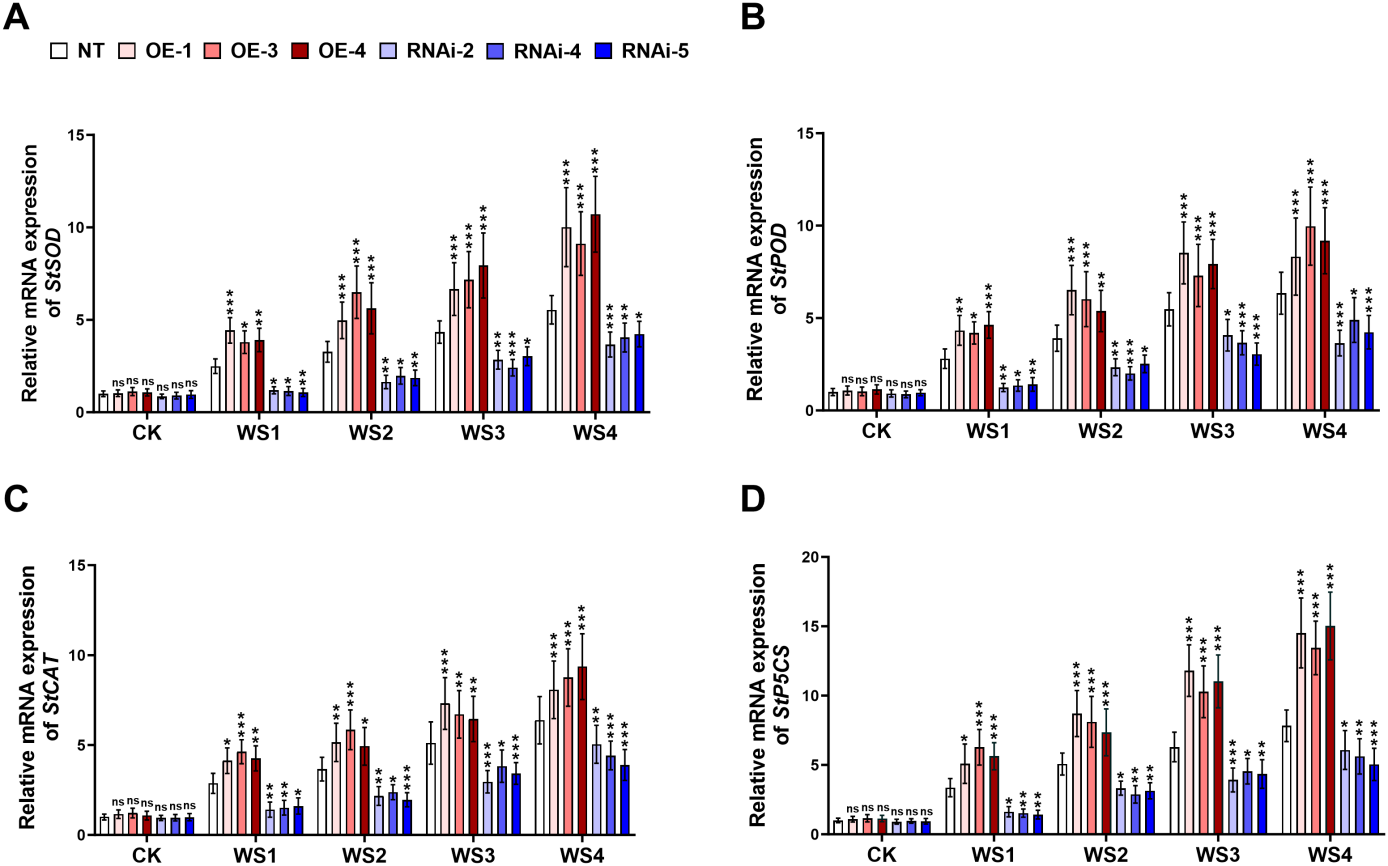
*StGATA14* modulates potato stress-responsive gene expression of cultivar *Atlantic*; **(A)** *StSOD*, **(B)** *StPOD*, **(C)** *StCAT*, and **(D)** *StP5CS*, after exposure to drought stress treatments (CK: 70–75%; WS1: 60–65%; WS2: 50–55%; WS3: 40–45%; WS4: 30–35%). NT, non-transgenic plants; OE, pBI121-EGFP-StGATA14-transgenic plants (OE-1, OE-3, and OE-5); RNAi, pART-StGATA14-RNAi-transgenic plants (RNAi-2, RNAi-4 and RNAi-5). The data are presented as mean ± standard deviation. Data are presented as mean ± standard deviation (n = 9). Statistical significance was determined by one-way ANOVA followed by Tukey’s multiple comparisons test, with “ns” indicating no significant difference, *P < 0.05, **P < 0.01, ***P < 0.001.

To further investigate the regulatory contribution of *StGATA14* to drought-responsive transcriptional networks, the expression profiles of several key stress-associated genes, *StSOD, StPOD, StCAT*, and *StP5CS*, were examined in *StGATA14*-OE, RNAi-knockdown, and NT potato lines exposed to varying degrees of water deficit. These genes were selected because of their established roles in antioxidant defense and osmotic regulation during abiotic stress. Under well-watered conditions (CK; 70–75% soil moisture), transcript levels of all four genes were comparable across OE, RNAi, and NT lines. This absence of significant variation indicates that modification of *StGATA14* expression does not alter the basal transcriptional activity of these genes in the absence of environmental stress, suggesting that *StGATA14* likely functions as a stress-responsive regulatory component rather than a constitutive activator of these pathways (**Figures 7A–D**).

When plants were subjected to mild drought stress (WS1; 60–65% soil moisture), genotype-dependent transcriptional differences became evident. The OE lines exhibited a significant increase in the transcript abundance of *StSOD* (**Figure 7A**), *StPOD* (**Figure 7B**), *StCAT* (**Figure 7C**), and *StP5CS* (**Figure 7D**) relative to NT lines, indicating that elevated *StGATA14* expression facilitates the early activation of genes involved in reactive oxygen species detoxification and osmotic adjustment. In contrast, RNAi lines displayed comparatively lower transcript levels, suggesting that reduced *StGATA14* expression limits the full induction of these protective genes during the initial phase of drought stress. As water limitation intensified to moderate (WS2; 50–55%) and severe (WS3; 40–45%) levels, the divergence among genotypes became more pronounced. OE (OE-1/3/5) lines consistently maintained higher transcript levels of all four genes compared with NT lines (**Figures 7A–D**), reflecting a sustained activation of antioxidant and osmoprotective mechanisms under increasing stress pressure. Conversely, RNAi lines (RNAi-2/4/5) showed markedly reduced expression of these genes, indicating that suppression of *StGATA14* compromises the maintenance of an effective drought-responsive transcriptional program. Under extreme drought conditions (WS4; 30–35% soil moisture), a general reduction in transcript accumulation was observed across all genotypes, likely reflecting the physiological constraints imposed by severe dehydration. Despite this overall decline, a clear genotype-dependent pattern persisted: OE lines retained relatively higher expression levels of *StSOD, StPOD, StCAT*, and S*tP5CS*, whereas RNAi lines exhibited the lowest transcript abundance (**Figures 7A–D**). In summary, the results of this study indicate that *StGATA14* positively regulates the transcript levels of key drought-responsive genes, including *StSOD, StPOD, StCAT*, and *StP5CS*. The altered expression patterns of these genes are consistent with the enhanced antioxidant capacity and osmotic adjustment observed in OE lines, suggesting that *StGATA14* may participate in the potato drought response by modulating the expression of these downstream genes.

### 3.7 *StGATA14* modulates gas exchange dynamics and intrinsic WUE across a drought intensity gradient

**Figure 8:**
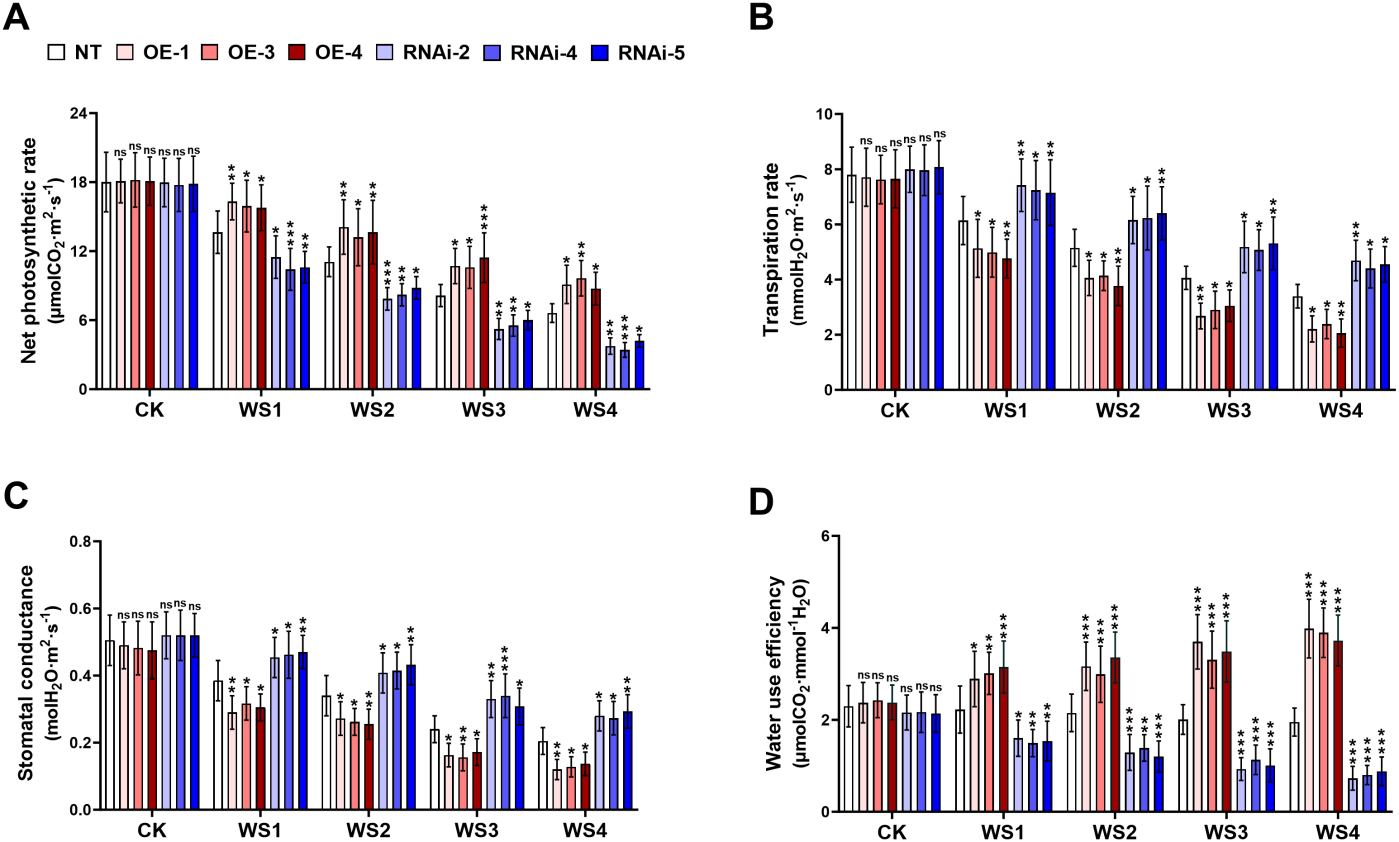
*StGATA14* confers potato gas exchange parameters of cultivar *Atlantic*; **(A)** net photosynthetic rate, **(B)** transpiration rate, **(C)** stomatal conductance, and **(D)** water use efficiency, after exposure to drought stress treatments (CK: 70–75%; WS1: 60–65%; WS2: 50–55%; WS3: 40–45%; WS4: 30–35%). NT, non-transgenic plants; OE, pBI121-EGFP-StGATA14-transgenic plants (OE-1, OE-3, and OE-5); RNAi, pART-StGATA14-RNAi-transgenic plants (RNAi-2, RNAi-4 and RNAi-5). The data are presented as mean ± standard deviation. Data are presented as mean ± standard deviation (n = 9). Statistical significance was determined by one-way ANOVA followed by Tukey’s multiple comparisons test, with “ns” indicating no significant difference, *P < 0.05, **P < 0.01, ***P < 0.001.

To assess the impact of *StGATA14-*mediated transcriptional regulation on photosynthetic performance under drought stress, key gas-exchange parameters, including net photosynthetic rate (Pn), transpiration rate (E), stomatal conductance (Gs), and intrinsic water use efficiency (WUE), were measured in *StGATA14*-OE, RNAi knockdown, and NT potato lines under well-watered control conditions (CK; 70–75% soil moisture) and progressive drought stress treatments (WS1–WS4). Under control conditions, no significant differences were observed among genotypes for Pn, E, Gs, or WUE, indicating that altered *StGATA14* expression does not affect gas-exchange characteristics in the absence of stress (**Figures 8A–D**). Under mild drought stress (WS1; 60–65% soil moisture), all genotypes showed reductions in gas-exchange parameters, consistent with the typical physiological response to limited water availability. However, the magnitude of these changes differed among genotypes. The OE lines maintained significantly higher Pn and WUE than NT plants (**Figures 8A–D**), while exhibiting relatively lower Gs and E under drought conditions (**Figures 8B, C**). This pattern suggests that *StGATA14*-OE enhances photosynthetic performance while limiting water loss through partial stomatal regulation, thereby improving water-use efficiency during early drought stress. In contrast, RNAi lines displayed markedly reduced Pn and WUE together with impaired stomatal regulation, indicating increased drought sensitivity. As drought stress intensified to moderate (WS2; 50–55% soil moisture) and severe (WS3; 40–45% soil moisture) levels, the physiological differences among genotypes became more pronounced. *StGATA14*-OE lines consistently maintained higher Pn and WUE compared with NT and RNAi plants, despite reduced Gs and E under water-deficit conditions (**Figures 8A–D**). These results indicate that OE plants were able to sustain relatively efficient photosynthesis while minimizing transpirational water loss, reflecting improved physiological adaptation to drought stress. In contrast, RNAi plants exhibited substantial declines in Pn, Gs, E, and WUE, suggesting impaired photosynthetic performance and reduced drought tolerance. Under extreme drought stress (WS4; 30–35% soil moisture), gas-exchange parameters declined markedly in all genotypes due to severe water stress. Nevertheless, OE lines retained comparatively higher Pn and WUE than NT and RNAi plants, while maintaining lower transpiration rates and stomatal conductance (**Figures 8A–D**). These findings further support the notion that *StGATA14* contributes to drought adaptation by optimizing the balance between photosynthetic assimilation and water conservation under limited water availability. Collectively, the results demonstrate that *StGATA14* positively regulates drought-responsive photosynthetic adaptation in potato by maintaining relatively stable photosynthetic activity while reducing excessive water loss, thereby improving intrinsic water-use efficiency under drought stress.

## 4 Discussion

GATA transcription factors have emerged as important regulators of plant adaptation to water deficit, with accumulating evidence from multiple species indicating their involvement in drought-responsive signaling networks (Schwechheimer et al., 2022; Hu et al., 2023; Aksoy et al., 2024; Alwutayd et al., 2026). Despite this progress, the functional characterization of GATA members in potato under water-limited conditions remains limited. This work aimed to address this gap by clarifying the function of *StGATA14* in drought stress response.

The conserved GATA zinc finger domain identified in StGATA14 and its orthologs highlights the evolutionary stability of this transcription factor family and supports its conserved DNA-binding function in plants (**Figure 2A**). Similar conservation of the cysteine-rich zinc finger motif has been widely reported for plant GATA proteins, which typically regulate gene expression through binding to GATA cis-elements in target promoters (Zhang et al., 2023). Consistent with these reports, our phylogenetic analysis showed that StGATA14 clusters closely with homologs from *Solanum lycopersicum* and *Solanum pennellii*, reflecting their close evolutionary relationship within the Solanaceae lineage (**Figure 2B**). Comparable phylogenetic grouping of GATA transcription factors among closely related species has also been observed in recent comparative analyses of plant GATA families (Kim, 2024). While our results agree with previous studies in demonstrating strong conservation of the zinc finger domain, minor sequence variations outside this region suggest potential functional diversification among species. The stress-induced expression pattern of *StGATA14* observed in this study suggests that it functions as an important regulator in drought-responsive signaling in potato. The stress-induced expression pattern of *StGATA14* observed in this study suggests that it functions as an important regulator in drought-responsive signaling in potato. Notably, *StGATA14* showed rapid and significant induction in the roots under all water-deficit conditions (WS1-WS4; **Figure 3**), indicating its possible involvement in early stress response in roots, the primary organs responsible for sensing soil water status. Similar tissue-specific expression patterns of GATA transcription factors have been reported in other plant species. For instance, genome-wide analysis in barley revealed that several *HvGATA* genes are differentially regulated under drought stress and exhibit organ-specific expression patterns, with some members showing preferential induction in roots (Zhang et al., 2025). Likewise, in *Arabidopsis*, GATA transcription factors have been implicated in abiotic stress signaling and regulation of stress-responsive gene networks that contribute to plant adaptation (Behringer and Schwechheimer, 2015). Studies in tomato also demonstrated that GATA genes display stress-inducible and tissue-dependent expression profiles, highlighting their role in integrating developmental and environmental signals (Reyes et al., 2004). While the conserved role of GATA transcription factors in stress responses is well established, the present study further reveals that *StGATA14* exhibits rapid, sustained, and strong induction in roots under progressive drought stress, suggesting its potential involvement in the early perception of drought signals within root tissues. It may subsequently regulate adaptive responses in aerial parts via long-distance signaling molecules, such as abscisic acid (ABA) and ROS (Davies and Zhang, 1991; Mittler et al., 2004). However, the hypothesis that *StGATA14* mediates root-based drought perception and systemic regulation currently lacks direct tissue-specific functional evidence, for example, from root-specific transgenic lines. This proposed regulatory mechanism requires further validation through subsequent tissue-specific genetic experiments.

The GATA family comprises nuclear-localized transcription factors that regulate gene expression by binding to GATA cis-elements, thereby controlling diverse processes, including nitrogen metabolism, light signaling, and abiotic stress responses, in plants (Gao et al., 2025). In line with the canonical behavior of transcription factors, subcellular localization analysis confirmed that StGATA14 is a nuclear protein. The StGATA14–GFP fusion construct, driven by the CaMV-35S promoter, displayed strong and exclusive fluorescence within the nucleus. In contrast, the free GFP control showed diffuse distribution across the cytoplasm, membrane, and nucleus (**Figure 4**). The nuclear localization of StGATA14 is consistent with the canonical feature of transcription factors, indicating its potential role in regulating downstream drought-responsive genes. However, it is important to emphasize that nuclear localization, while necessary, is not sufficient for transcription factor activity (Latchman, 1997). The direct DNA-binding specificity and transcriptional activation function of StGATA14 remain to be experimentally demonstrated. This finding is consistent with previous work in rice, where the precise co-localization of OsGATA16-GFP with DAPI-stained nuclei confirmed its nuclear localization and supported its role as a transcriptional regulator of cold stress responses (Zhang et al., 2021). While the present study establishes the subcellular basis for StGATA14 function, it does not directly confirm its DNA-binding specificity or transcriptional activation capability. Therefore, further experiments, including yeast one-hybrid assays, electrophoretic mobility shift assays (EMSA), chromatin immunoprecipitation (ChIP), and dual-luciferase reporter assays, will be necessary to determine whether StGATA14 directly binds to the promoters of drought-responsive genes and to elucidate the precise molecular mechanisms underlying its role in drought stress tolerance.

Growth-related traits, including plant height, leaf area, total biomass, and root-to-shoot ratio, are primary integrative indicators of plant performance under drought stress (Hussain et al., 2024). Water deficit generally suppresses shoot elongation and leaf expansion to minimize transpirational loss, while stimulating root proliferation to improve soil water acquisition. This reorganization of assimilates reflects an adaptive balance between resource conservation and continued growth, ultimately influencing drought resilience and yield stability (Zahedi et al., 2025). *StGATA14*-overexpressing potato lines (cv. *Atlantic*) (OE-1/3/5) exhibited significantly enhanced growth under various water stress conditions, viz., WS1 (60–65% soil moisture), WS2 (50–55%), WS3 (40–45%), and WS4 (30–35%). OE lines maintained greater plant height, plant and root fresh and dry biomass, and overall vigor compared with NT controls, whereas RNAi lines (RNAi-2/4/5) showed pronounced growth inhibition under identical stress regimes (**Supplementary Table 3**, **Figure 5**). Notably, the simultaneous improvement in both plant and root biomass in OE lines suggests that *StGATA14* does not merely shift biomass allocation toward root elongation, a typical drought response, but instead sustains whole-plant growth capacity under water limitation. Comparative evidence from other GATA family members supports a conserved role in growth maintenance under abiotic stress while revealing stress-specific mechanistic distinctions. In rice, *OsGATA8* overexpression significantly enhanced biomass accumulation under salinity stress and sustained growth under non-stress conditions. This effect was associated with regulation of chlorophyll biosynthesis and ROS-scavenging genes, improved water status, and approximately 46% higher yield relative to wild type under stress (Nutan et al., 2020). Similarly, under heat stress, *StGATA2-*overexpressing potato lines maintained increased plant height and plant/root biomass, whereas RNAi lines exhibited reduced growth compared with NT lines (Zhu et al., 2024). Similarly, PdGNC, a GATA TF from poplar, improved photosynthetic capacity and biomass accumulation under low-nitrogen conditions by increasing chloroplast number and chlorophyll levels, thereby promoting nitrogen utilization efficiency (An et al., 2014). Across these studies, a clear similarity emerges: overexpression of *GATA* genes consistently promotes biomass retention and growth stability under diverse abiotic stresses, including drought, salinity, heat, and nitrogen deficiency. In all cases, improved performance is associated with enhanced physiological homeostasis, particularly maintenance of chlorophyll content, growth and biomass, water status, and oxidative balance. However, key differences are evident in the stress context and dominant regulatory mechanisms. Under salinity (*OsGATA8*), biomass enhancement is closely linked to ion homeostasis and chlorophyll biosynthesis pathways. Under heat stress (*StGATA2*), improved growth is associated with thermal protection and sustained metabolic activity, and *PdGNC* enhances nitrogen efficiency through chloroplast development. In contrast, *StGATA14*-mediated drought tolerance appears to emphasize coordinated maintenance of water status, photosynthetic capacity, and balanced shoot–root development without excessive morphological trade-offs. Thus, GATA TFs represent conserved yet functionally specialized regulators with significant potential for improving crop resilience and productivity under multiple environmental constraints.

GATA transcription factors have emerged as important regulators of abiotic stress tolerance through coordinated modulation of antioxidant defense, osmotic adjustment, and stress-responsive gene expression (Rehaman et al., 2025; Abd-El-Aty et al., 2024). In the present study, *StGATA14*-OE potato lines displayed enhanced drought tolerance characterized by reduced oxidative damage, as evidenced by lower H_2_O_2_ and MDA accumulation (**Figures 5A, B**), together with increased activities of antioxidant enzymes (SOD, CAT, and POD; **Figures 5F–H**) and elevated levels of osmoprotectants, including proline, soluble sugars, and soluble proteins (**Figures 5C–E**). Consistently, the expression levels of stress-responsive genes associated with antioxidant defense and osmotic regulation, including *StP5CS, StCAT, StPOD*, and *StSOD*, were significantly upregulated in OE lines (**Figures 7A–D**), whereas RNAi plants exhibited opposite trends, confirming that *StGATA14* positively contributes to drought adaptation in potato. These findings are consistent with previous reports showing that *SlGATA17* improves drought tolerance in tomato by enhancing ROS scavenging and osmotic adjustment mechanisms (Zhao et al., 2021). Similar stress-protective functions have also been reported for other potato GATA genes, including *StGATA2* and *StGATA12*, which participate in heat and salt stress tolerance, respectively (Zhu et al., 2023, 2024). Collectively, these observations suggest that GATA transcription factors may function as conserved regulators of abiotic stress adaptation, although individual members likely exhibit functional specialization under distinct stress conditions. Notably, *StGATA14* appears to play a more prominent role in drought-associated antioxidant and osmotic regulation compared with previously characterized GATA members primarily involved in nutrient metabolism or other abiotic stresses. For example, the *Arabidopsis* GATA factor GNC mainly regulates carbon and nitrogen metabolism and chlorophyll biosynthesis under nitrate-responsive conditions (Bi et al., 2005), whereas *StGATA14* functions predominantly under water-deficit stress to maintain cellular redox balance and osmotic homeostasis. These differences indicate potential functional diversification within the GATA transcription factor family during plant stress adaptation.

However, the observed physiological and transcriptional changes strongly support the involvement of *StGATA14* in drought tolerance, the present study provides primarily correlative evidence regarding downstream gene regulation. While the increased expression of *StP5CS, StCAT, StPOD*, and *StSOD* in transgenic lines suggests that *StGATA14* may regulate antioxidant and osmotic stress-responsive pathways, direct transcriptional regulation was not experimentally validated. Promoter-binding assays such as EMSA, ChIP-qPCR, yeast one-hybrid, or dual-luciferase assays were not performed in this study. Therefore, it remains unclear whether StGATA14 directly binds to the promoters of these target genes or indirectly regulates their expression through interaction with other transcriptional regulators or signaling pathways. Future studies integrating promoter interaction analyses and protein–DNA binding assays will be essential to elucidate the precise molecular mechanism underlying *StGATA14-*mediated drought tolerance in potato.

Plants alleviate drought-induced damage through tightly coordinated morphological and physiological adjustments, with the maintenance of leaf RWC, chlorophyll stability, stomatal regulation, and gas exchange performance being the core determinants of adaptive capacity (Haghpanah et al., 2024; Zahedi et al., 2025). In this study, under all drought stress treatments (WS1-WS4), *StGATA14-*OE potato lines maintained significantly higher Pn and WUE, along with lower Gs and E, compared to NT plants, which exhibited the opposite trends (**Figure 8**). Physiological assessment revealed that the OE lines employed a synergistic drought resistance strategy characterized by “moderate stomatal closure coupled with sustained photosynthetic efficiency” under drought conditions. Specifically, *StGATA14*-OE induced moderate stomatal closure, effectively reducing transpirational water loss and thereby maintaining superior leaf water status. Concurrently, the enhanced antioxidant system in OE lines, evidenced by elevated activities of SOD, CAT, and POD, alongside reduced levels of H_2_O_2_ and MDA, protected chloroplast structure and the integrity of the photosynthetic electron transport chain. Furthermore, improved osmotic adjustment, achieved through the accumulation of proline, soluble sugars, and soluble proteins, contributed to the maintenance of cell turgor and enzyme activity. The synergistic action of these adaptations ensured efficient CO_2_ assimilation could proceed despite partial stomatal limitation. The enhancement in WUE stemmed from a proportionally greater reduction in E than in Pn. In the OE lines, moderate stomatal closure markedly decreased E, while the protection of photosynthetic machinery maintained a relatively high Pn, resulting in greater carbon assimilation per unit of water transpired. This strategy, which conserves water without incurring substantial carbon loss, is more efficient for drought resistance than severe stomatal closure alone, favoring the maintenance of growth and biomass accumulation under water deficit. Similar regulatory patterns have been documented for other GATA transcription factors. For instance, in poplar, overexpression of *PdGNC* enhances drought tolerance by modulating stomatal movement through *PdHXK*1-mediated accumulation of nitric oxide (NO) and H_2_O_2_ in guard cells, leading to reduced stomatal aperture, improved WUE, and enhanced drought resistance, whereas CRISPR-Cas9 knockout mutants exhibit significantly compromised drought tolerance (Shen et al., 2021). *PdGNC* also promotes plant growth by coordinately regulating photosynthetic electron transport, carbon assimilation, nitrogen uptake, and cell division (An et al., 2020). Similarly, in potato, *StGATA2* overexpression enhances thermotolerance by reducing stomatal size, increasing photosynthetic efficiency, and lowering transpiration rate (Zhu et al., 2023). In summary, these studies support a conserved role for GATA transcription factors in optimizing stomatal behavior, photosynthetic performance, and water balance under abiotic stress, although the underlying regulatory mechanisms may be stress-specific. In the context of our findings, *StGATA14*-mediated drought resistance is primarily associated with sustained gas exchange capacity and efficient coordination between stomatal conductance and photosynthetic carbon assimilation. However, whether *StGATA14* directly regulates guard cell signaling pathways or stomatal movement remains to be elucidated and warrants further investigation.

Despite establishing *StGATA14* as a key coordinator of antioxidant defense and osmotic homeostasis in drought tolerance, several limitations warrant acknowledgment in this study. The use of relative qRT-PCR precludes direct comparison of absolute transcript levels across tissues. Future studies employing absolute quantification (e.g., via an RNA standard curve) or spatial techniques (e.g., RNA *in situ* hybridization) are needed to precisely define the tissue-specific expression profile of *StGATA14*. The experiments were conducted exclusively under controlled greenhouse conditions, which may not fully replicate the dynamic and multifactorial nature of field environments. Moreover, the investigation was confined to a single potato cultivar (*Atlantic*), necessitating validation across diverse genetic backgrounds to ascertain the broader applicability of the findings. Additionally, the downstream molecular mechanisms, including direct target genes, upstream regulators, and post-translational modifications, remain to be comprehensively elucidated. Future application of advanced technologies such as CRISPR-based genome editing, chromatin immunoprecipitation sequencing (ChIP-seq), transcriptomics, proteomics, and epigenetic technology will be essential to delineate the hierarchical regulatory networks and precise cellular functions of *StGATA14* in mediating drought adaptation.

## 5 Conclusion

This study identifies *StGATA14* as a positive regulator conferring drought tolerance in potato. Its expression is rapidly and strongly induced by water deficit, with predominant upregulation in roots, indicating its involvement in the drought stress response. Functional validation via transgenic approaches confirmed that *StGATA14*-OE enhances drought resilience, whereas RNAi-mediated knockdown increases sensitivity. The enhanced stress tolerance observed in overexpression lines correlates with increased photosynthetic efficiency, strengthened antioxidant defenses, and improved osmotic regulation.

## Data Availability

The original contributions presented in the study are included in the article/**Supplementary Material**. Further inquiries can be directed to the corresponding authors.
