# Supplementary material for: StGATA14 coordinates antioxidant defense and osmotic homeostasis to enhance drought tolerance in potato
Source: Front Plant Sci. 2026 Jun 23;17:1858357. doi: 10.3389/fpls.2026.1858357 (PMC13337500; doi:10.3389/fpls.2026.1858357)
Supplement: Supplementary file 1 [file SupplementaryFile1.docx]

**Title:  *StGATA14* coordinates antioxidant defense and osmotic homeostasis to enhance drought tolerance in potato**

**Supplementary Materials**

1. **Supplementary Figures**


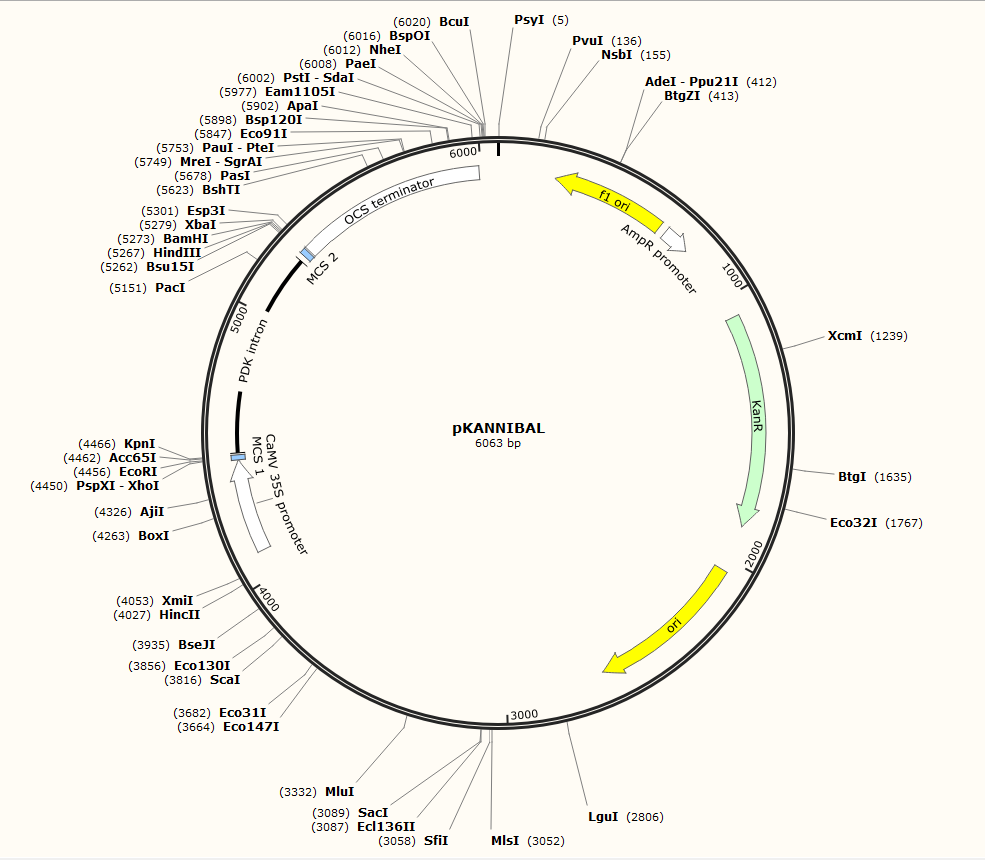


**Figure S1. Restriction Enzyme Map of the pKANNIBAL RNAi Intermediate Vector.**  pKANNIBAL is a widely used dedicated intermediate vector for RNA interference (RNAi) studies in plant functional genomics, with a full length of 6063 bp. This vector adopts the classic intron-spliced hairpin RNA (ihpRNA) design framework, and its core expression elements include the constitutive cauliflower mosaic virus 35S promoter (CaMV 35S promoter), two oppositely oriented multiple cloning sites (MCS1 and MCS2), the pyruvate dehydrogenase kinase intron (PDK intron), and the octopine synthase terminator (OCS terminator). By inserting the sense and antisense specific fragments of the target gene into MCS1 and MCS2 respectively, a hairpin RNA structure with high silencing efficiency can be transcribed in plant cells. The vector also carries the ampicillin resistance gene (*AmpR*) and kanamycin resistance gene (*KanR*) for selection in Escherichia coli transformation, and contains a bacterial origin of replication (*ori*) to ensure stable replication of the plasmid in prokaryotic systems. In this study, a 300 bp specific coding region fragment of the *StGATA14* gene was cloned into the MCS1 and MCS2 sites in the sense and antisense orientations, respectively, to construct the pHAN-StGATA14-RNAi intermediate vector. Subsequently, the complete RNAi expression cassette was excised using restriction endonucleases and subcloned into the pART binary plant expression vector, which was ultimately used for *Agrobacterium*-mediated genetic transformation of potato. The recognition sites of major restriction endonucleases on the vector and their corresponding base positions (unit: bp) are indicated in the figure.


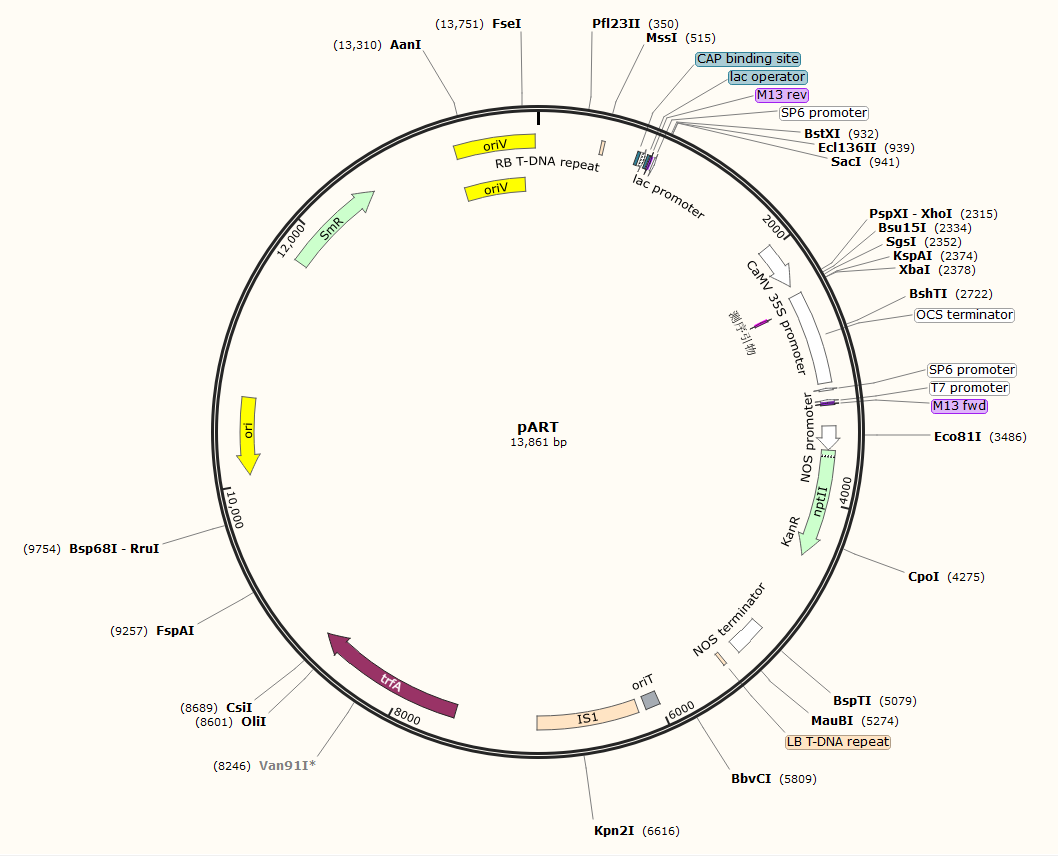


**Figure S2. Restriction map of the pART binary plant expression vector.** pART is a widely used broad-host-range binary vector for Agrobacterium-mediated plant genetic transformation, with a total length of 13,861 bp. The vector comprises two core functional regions: (1) The **T-DNA transfer region**, delineated by the right border (RB T-DNA repeat) and left border (LB T-DNA repeat), is the fragment specifically integrated into the plant genome during Agrobacteriuminfection. Within the T-DNA, the constitutive cauliflower mosaic virus 35S promoter (CaMV 35S promoter), a multiple cloning site (MCS), and the octopine synthase terminator (OCS terminator) are included for constitutive expression of the gene of interest in plants. It also contains the neomycin phosphotransferase II gene (nptII) driven by the nopaline synthase promoter (NOS promoter), conferring kanamycin resistance to transgenic plants for selecting positive transformants. (2) The **prokaryotic replication and selection region** contains a broad-host-range origin of replication (oriV), an origin of transfer (oriT), and the replication initiation protein gene (trfA), ensuring stable replication and conjugal transfer in both Escherichia coli and Agrobacterium tumefaciens. The streptomycin resistance gene (SmR) is used for selection in prokaryotic hosts. Additionally, the vector is equipped with a lac promoter, M13 forward/reverse primer binding sites, and SP6/T7 promoters to facilitate cloning, sequencing, and in vitro transcription of the insert. In this study, the complete RNAi expression cassette, excised from the intermediate vector pHAN-StGATA14-RNAi, was inserted into the MCS of pART to construct the final plant transformation vector, pART-StGATA14-RNAi. The map indicates the recognition sites and corresponding nucleotide positions (in bp) of the major restriction enzymes on the vector.


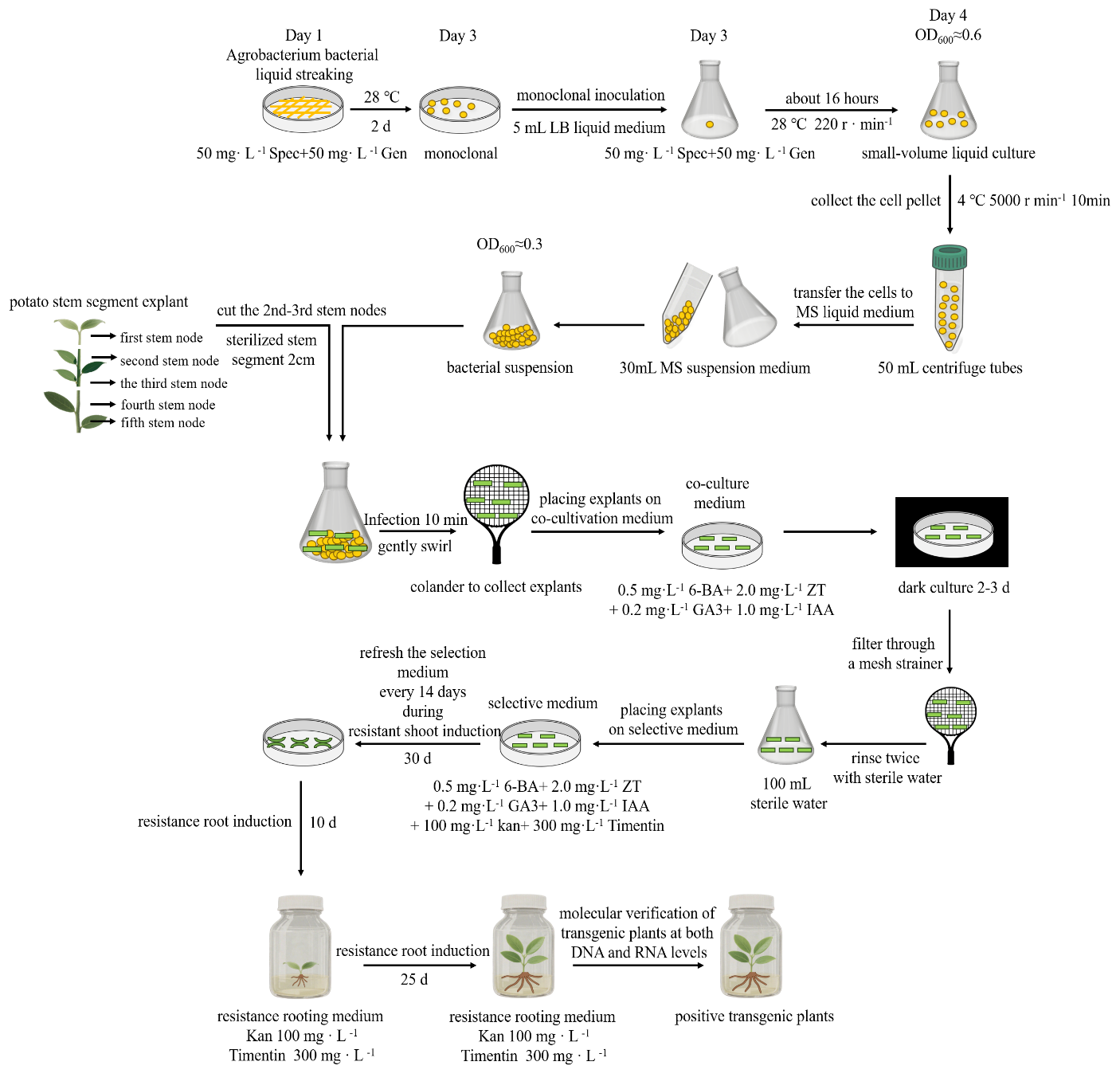


**Figure S3**. Schematic flowchart showing major steps involved in the generation of transgenic potato plants using *Agrobacterium*-mediated transformation methods.

**
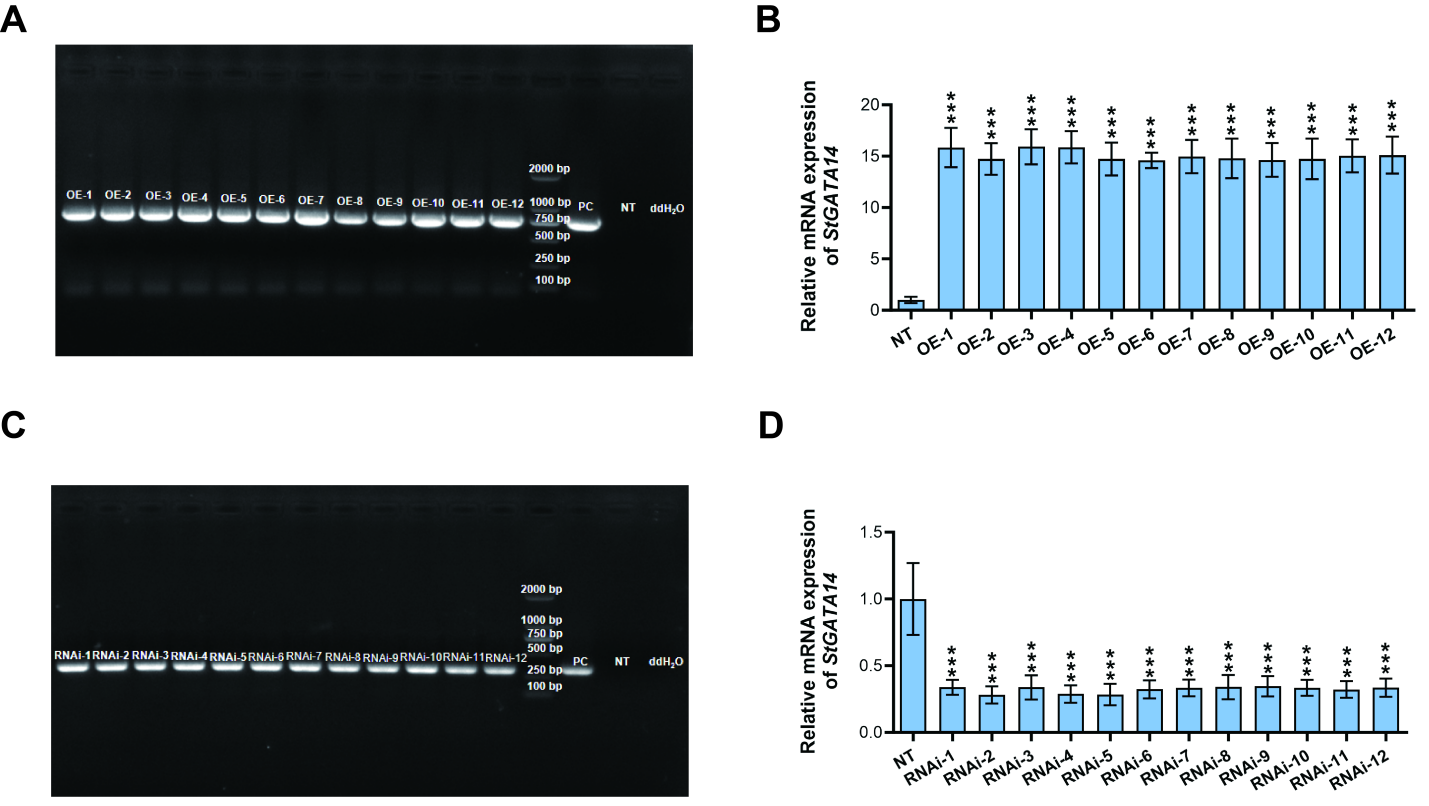
**

**Figure S4.** Molecular characterization of transgenic potato lines for *StGATA14*. (**A**) PCR amplification of the specific fragment in OE lines; (**B**) Relative *StGATA14* mRNA expression levels in OE lines of the *‘Atlantic’* cultivar; (**C**) PCR amplification of the specific fragment in RNAi lines; (**D**) Relative *StGATA14* mRNA expression levels in RNAi lines of the *‘Atlantic’* cultivar. In panels A and C, OE represents pBI121‑EGFP‑StGATA14 overexpression transgenic lines (OE‑1 to OE‑12), and RNAi represents pART‑StGATA14‑RNAi interference transgenic lines (RNAi‑1 to RNAi‑12); PC denotes plasmid positive control, NT indicates non‑transgenic plants, and ddH₂O serves as the negative control. In panels B and D, NT refers to non‑transgenic lines; OE represents pBI121‑EGFP‑StGATA14 overexpression transgenic lines (OE‑1 to OE‑12); RNAi indicates pART‑StGATA14‑RNAi interference transgenic lines (RNAi‑1 to RNAi‑12). Data are presented as mean ± standard deviation. Statistical significance was determined using ordinary two‑way ANOVA followed by Tukey’s multiple comparisons test with a sample size of n = 3, and ****P < 0.001*.

1. **Supplementary Tables**

**Table S1. Sequences of primers used in the present study**

| **Gene ID** | **Gene** | **Forward (5’-3’)** | **Reverse (5’-3’)** |
| --- | --- | --- | --- |
| **Primer required for overexpression：** | | | |
| XM_006346941.2 | *StGATA14* | CTCGACATGGATTACTCCGGCAACTGT | GTCGACAAAACTCTGAACCGGCGGACCCG |
| **Primer required for RNA interference expression：** | | | |
| XM_006346941.2 | *StGATA14* | GATTACTCCGGCAACTGTCAA | TGAACTTCGAGAGCTTTTTCCGGTA |
| **Primer required for subcellular localization：** | | | |
| XM_006346941.2 | *StGATA14* | CTCGAGATGTCTATGAAAAATACCCAACAAG | GTCGACACAAGTTGAAATCATAGAAGCTAAACCGG |
| **Primers required for qRT-PCR:** | | | |
| XM_006347752.2 | *StEf1α* | GGTTGTATCTCTTCCGATAAAGGC | GGTTGTATCTCTTCCGATAAAGGC |
| XM_015308529.1 | *StP5CS* | TGCAATGCAATGGAAACGCT | ACAATTTCCACGGTGCAAGC |
| AY442179 | *StCAT* | CCATGCTGAGGTGTATCCTATTC | CCTTTCTCCTGGTTGCTTGA |
| AF354748 | *StSOD* | CATTGGAAGAGCTGTTGTTGTT | ATCCTTCCGCCAGCATTT |
| XM_006362636.2 | *StPOD* | AGATGTTGTGGCCATGTCTGG | GCTTGTGTTGAAGGATGGAGC |
| XM_006346941.2 | *StGATA14* | GAACTCAGTCTTCCTGGGGC | TCGTTCGGCACTAAACGGAA |

The above-mentioned genes can be directly accessed through the National Center for Biotechnology Information (NCBI database) ([https://www.ncbi.nlm.nih.gov/guide/](https://www.ncbi.nlm.nih.gov/guide/)).

**Table S2.** **Protein IDs of GATA in different plant species.**

| **Plant species** | **Protein** | **Protein ID** |
| --- | --- | --- |
| *Arabidopsis thaliana* | AtGATA24 | NP_566676.1 |
| *Solanum pennellii* | SpGATA5 | XP_015065182.1 |
| *Solanum tuberosum* | StGATA14 | XP_006347003.1 |
| *Solanum lycopersicum* | SlGATA5 | XP_004233547.1 |
| *Nicotiana sylvestris* | NsGATA5 | XP_009761911.1 |
| *Sesamum indicum* | SiGATA5 | XP_011100952.1 |
| *Ipomoea triloba* | ItGATA5 | XP_031115062.1 |
| *Capsicum annuum* | CaGATA5 | XP_016560558.1 |
| *Vitis vinifera* | VvGATA5 | RVX16781.1 |
| *Prunus avium* | PaGATA5 | XP_021816039.1 |
| *Capsicum chinense* | CcGATA5 | PHU27103.1 |
| *Manihot esculenta* | MeGATA5 | XP_021602140.1 |

The amino acid sequence of this protein can be directly accessed and retrieved via the National Center for Biotechnology Information (NCBI database) (<https://www.ncbi.nlm.nih.gov/guide/>).

**Table S3. Effects of *StGATA14* on potato growth indicators under drought stresses.**

| **Salt stress** | **Genotype** | **Plant height (cm)** | **Fresh weight (g)** | **Dry weight (g)** | **Root fresh weight (g)** | **Root dry weight (g)** |
| --- | --- | --- | --- | --- | --- | --- |
| **CK** | NT | 49.70±5.10 | 180.97±18.57 | 30.71±5.78 | 19.37±1.98 | 3.01±0.25 |
|  | OE-1 | 50.50±5.90ns | 185.25±17.79ns | 31.38±4.07ns | 19.65±1.83ns | 3.08±0.35ns |
|  | OE-3 | 50.80±4.90ns | 181.32±18.12ns | 31.23±6.12ns | 19.70±2.19ns | 3.08±0.43ns |
|  | OE-4 | 49.80±5.00ns | 183.84±21.55ns | 30.66±5.18ns | 19.23±1.87ns | 3.01±0.38ns |
|  | RNAi-2 | 47.80±7.10ns | 173.27±24.73ns | 30.28±5.63ns | 18.84±2.00ns | 2.92±0.33ns |
|  | RNAi-4 | 45.20±6.20ns | 182.93±23.39ns | 28.33±4.17ns | 17.77±1.96ns | 2.78±0.43ns |
|  | RNAi-5 | 46.20±5.70ns | 176.84±22.69ns | 29.04±4.78ns | 18.56±1.86ns | 2.85±0.36ns |
| **WS1** | NT | 43.60±5.50 | 146.73±19.09 | 23.12±4.63 | 15.34±1.67 | 2.31±0.46 |
|  | OE-1 | 46.90±5.90ns | 173.81±22.27* | 28.80±3.18* | 17.59±1.84* | 2.71±0.45* |
|  | OE-3 | 47.80±6.10ns | 167.60±24.91ns | 29.21±3.47** | 18.78±2.08*** | 2.94±0.32*** |
|  | OE-4 | 46.10±6.90ns | 170.87±21.58* | 27.99±4.27* | 17.78±2.82* | 2.79±0.43** |
|  | RNAi-2 | 32.50±5.80* | 120.41±22.70* | 17.07±3.43** | 11.04±2.23*** | 1.61±0.33*** |
|  | RNAi-4 | 37.00±7.00ns | 105.59±18.79*** | 19.21±4.43ns | 13.12±1.09* | 1.77±0.35** |
|  | RNAi-5 | 35.60±6.80ns | 115.55±22.13** | 17.44±2.84* | 12.35±2.42** | 1.87±0.19* |
| **WS2** | NT | 32.20±4.10 | 118.00±14.53 | 19.04±2.69 | 12.72±1.16 | 1.98±0.18 |
|  | OE-1 | 43.20±5.30* | 163.36±24.13*** | 25.87±4.23** | 16.43±2.58*** | 2.59±0.32*** |
|  | OE-3 | 44.70±6.60** | 153.64±21.87*** | 27.02±6.01*** | 15.17±1.73* | 2.50±0.42** |
|  | OE-4 | 43.90±6.00** | 144.30±19.91* | 24.10±4.03* | 14.91±1.86* | 2.38±0.37* |
|  | RNAi-2 | 20.60±3.90* | 90.81±13.50* | 13.62±4.10* | 8.26±1.67*** | 1.50±0.31** |
|  | RNAi-4 | 21.10±4.70* | 72.11±14.44*** | 12.34±2.80** | 9.38±2.22*** | 1.31±0.21*** |
|  | RNAi-5 | 19.70±3.40** | 80.37±12.37*** | 11.12±2.96*** | 7.47±1.53*** | 1.17±0.24*** |
| **WS3** | NT | 29.20±5.70 | 99.46±19.30 | 15.26±3.08 | 10.42±0.96 | 1.68±0.27 |
|  | OE-1 | 39.90±5.50* | 132.29±25.56** | 21.87±4.26** | 12.62±1.53* | 2.08±0.43* |
|  | OE-3 | 40.20±6.90* | 123.58±20.62* | 20.05±5.15* | 13.24±2.49** | 2.17±0.27** |
|  | OE-4 | 41.10±7.90** | 140.89±27.49*** | 23.10±5.50*** | 13.83±2.33*** | 2.24±0.38** |
|  | RNAi-2 | 18.60±3.40* | 57.39±13.36*** | 9.79±2.49* | 6.47±0.77*** | 1.08±0.24*** |
|  | RNAi-4 | 18.20±3.50* | 50.21±16.20*** | 8.46±2.18** | 5.86±1.30*** | 0.98±0.20*** |
|  | RNAi-5 | 17.40±4.00** | 67.28±10.65** | 7.44±1.68*** | 5.42±1.08*** | 0.91±0.28*** |
| **WS4** | NT | 24.90±6.30 | 76.80±15.29 | 11.65±2.84 | 5.87±0.61 | 1.21±0.13 |
|  | OE-1 | 36.80±8.30** | 106.13±25.39** | 19.62±2.48*** | 8.09±1.80* | 1.61±0.40* |
|  | OE-3 | 36.20±8.90* | 111.22±23.35** | 18.05±3.21** | 10.47±1.91*** | 1.78±0.20** |
|  | OE-4 | 35.40±5.00* | 100.80±18.89* | 17.06±3.78* | 9.02±2.03** | 1.89±0.38*** |
|  | RNAi-2 | 13.90±4.40* | 32.26±08.08*** | 5.89±1.57* | 3.14±0.48** | 0.41±0.07*** |
|  | RNAi-4 | 13.20±4.10** | 44.82±07.78** | 3.88±0.65*** | 2.46±0.65*** | 0.55±0.15*** |
|  | RNAi-5 | 14.40±4.30* | 37.34±07.28*** | 4.87±1.21** | 3.68±0.65* | 0.61±0.14*** |

Phenotypes of potato, such as plant height, fresh weight, dry weight, root fresh weight, and root dry weight, were analyzed. Data are presented as mean ± standard deviation (n = 9). Statistical significance was determined by one-way ANOVA followed by Tukey’s multiple comparisons test, with “ns” indicating no significant difference, **P* < 0.05, ***P* < 0.01, ****P* < 0.001. Soil water content in the pots was remained at 70–75% in control group (CK), 60-65% in water stress 1 group (WS1), 50-55% in WS2, 40-45% in WS3, and 30-35% in WS4. Soil water content was monitored at 10:00 and 16:00 every day using TDR-300 sensors (Spectrum R, Aurora, IL, USA).

**Supplementary Data 1**

1. **Supplementary data 1**
   1. **Assessment of physiological indicators**

**3.1.1. Activity of POD**

POD activity was determined according to a previously described method (Li, 2000) with minor modifications. Briefly, 5.0 g of leaves were homogenized in 10 mL of phosphate-buffered saline (PBS). The homogenate was centrifuged at 3000 × g for 10 min, and the supernatant was collected, transferred into a 25 mL Erlenmeyer flask, and diluted with PBS. Then, 0.1 mL of the extracted solution was incubated with a reaction mixture containing 2.9 mL of 0.05 mol/L PBS, 1.0 mL of 2% H₂O₂, and 1.0 mL of 0.05 mol/L guaiacol at 37 °C for 15 min. The reaction was terminated by adding 2.0 mL of trichloroacetic acid (TCA). After filtration, the absorbance of the mixture was measured at 470 nm. An extracted solution boiled for 5 min was used as the control.

**3.1.2. Activity of CAT**

CAT activity was determined according to a previously described method (Li, 2000) with minor modifications. Briefly, 2.5 g of leaves were homogenized in 25 mL of phosphate buffered saline (pH7.8), and the supernatant was collected by centrifugation at 2486.4 × g for 15 min, followed by incubation with 2.5 mL of 0.1 M H_2_O_2_ at 30 ◦C for 10 min. The reaction was terminated by adding 2.5 mL of 10% H_2_SO_4_. The content of CAT was examined by 0.1 M KMnO_4_ titration in the presence of H_2_SO_4_. The extracted solution boiled for 5 min was used as a control.

**3.1.3. Activity of SOD**

SOD activity was determined according to a previously described method (Li, 2000) with minor modifications. Briefly, 0.5 leaves were homogenized in phosphate buffered saline and 5 mL mixture was obtained. The supernatant was collected by centrifugation at 155.4 × g for 20 min. The extract (0.05 mL) was incubated with the chromogenic reagent consisting of 1.5 mL of 0.05 mol/L phosphate buffered saline, 0.3 mL of 130 mM methionine, 0.3 mL of 750 μM nitroblue tetrazolium, 0.3 mL of 100 μM EDTA-Na_2_, 0.3 mL of 20 μM riboflavin, and 0.25 mL H_2_O under 4000 Lux for 20 min. The mixture was maintained in the dark as the control. The absorbance was examined at 560 nm.

- - 1. **H_2_O_2_ content**

The hydrogen peroxide (H₂O₂) content was determined according to the method described by Loreto and Velikova (2001) with minor modifications. Briefly, fresh leaf tissue (0.3 g) was homogenized in 5% (w/v) trichloroacetic acid (TCA), followed by centrifugation at 12,000 × g for 15 minutes. The supernatant was then reacted with 0.5 M potassium phosphate buffer (pH 7.0) and 1 M potassium iodide (KI). Absorbance was measured at 390 nm, and the H₂O₂ concentration was expressed as nanomoles per gram fresh weight (nmol·g⁻¹ FW).

- - 1. **Proline content determination**

Proline content in leaves was determined using a slightly modified protocol based on the method described by Bates et al. (1973). The procedures for proline extraction and quantification were as follows: Fresh leaf tissue (0.3 g) was homogenized in 3 mL of 3% (w/v) sulfosalicylic acid. The homogenate was centrifuged at 10,000 × g for 20 minutes at 4 °C to remove cellular debris. A 0.2 mL aliquot of the resulting supernatant was mixed with 0.4 mL of distilled water and 2 mL of reagent mixture (prepared by combining 30 mL glacial acetic acid, 20 mL distilled water, and 0.5 g ninhydrin). The reaction tubes were sealed and incubated in a boiling water bath for 1 hour. After cooling, 6 mL of toluene was added to extract the chromophore. Absorbance was immediately measured at 520 nm. Proline concentration was determined using a standard curve and expressed on a fresh weight basis as μmol proline per gram fresh weight (μmol proline·g⁻¹ FW).

- - 1. **MDA content determination**

The content of malondialdehyde (MDA), an indicator of lipid peroxidation, was evaluated by measuring thiobarbituric acid reactive substances (TBARS) according to the method of Heath and Packer (1968) with modifications. Leaf samples (0.5 g) from transgenic and non-transgenic potato plants were homogenized in a solution containing 20% (w/v) trichloroacetic acid (TCA) and 0.5% (w/v) thiobarbituric acid (TBA). The homogenate was incubated at 95 °C for 30 minutes, and the reaction was terminated on ice. The samples were then centrifuged at 12,000 × g for 10 minutes, and the resulting supernatant was used to measure absorbance at 532 nm and 600 nm. The nonspecific absorbance at 600 nm was subtracted from the absorbance at 532 nm, and the MDA concentration was calculated using the extinction coefficient of MDA (155 mM⁻¹·cm⁻¹).

- - 1. **Soluble sugar content**

The soluble sugar content in potato plants was determined according to the method described by Dubois et al. (1956). The specific procedures were as follows: Leaves (0.5 g) from independent transgenic and non-transgenic plants were ground in liquid nitrogen, followed by extraction with 4 mL of 80% (v/v) ethanol at 70 °C for 30 minutes. The extract was centrifuged at 8000 rpm for 10 minutes to separate the phases, and the aqueous phase was collected. The sugar content in the aqueous extract was measured using the phenol–sulfuric acid method.

- - 1. **Soluble protein content**

The soluble protein content in potato plants was determined according to the method described by Bradford (1976). Six test tubes were prepared, each containing 0–1.0 mL of standard protein solution (prepared by dissolving 25 mg bovine serum albumin in water, diluted to 100 mL; 40 mL of this solution was then diluted to 100 mL to obtain a 100 μg·mL⁻¹ working solution). Distilled water was added to each tube to adjust the final volume to 1.0 mL, resulting in protein contents ranging from 0 to 100 μg. Then, 5 mL of Coomassie Brilliant Blue solution (prepared by dissolving 100 mg Coomassie Brilliant Blue in 50 mL of 90% ethanol, adding 100 mL of 85% phosphoric acid, and diluting to 1 L with distilled water) was added to each tube. After thorough mixing, the solutions were incubated for 2 minutes, and absorbance was measured at 595 nm to generate the standard curve. A leaf sample (0.5 g) was homogenized with 2 mL of distilled water, followed by rinsing with 6 mL of distilled water. The homogenate was centrifuged at 4000 rpm for 10 minutes, and the supernatant was diluted to a final volume of 10 mL. A 1.0 mL aliquot of the sample solution was mixed with 5 mL of the dye reagent, incubated for 2 minutes, and absorbance was measured at 595 nm. The protein content (C) was determined from the standard curve. The protein content in the sample (mg·g⁻¹) is calculated using the following formula:

$$\text{Protein content}\text{ }\text{(}\text{mg}\text{/g }\text{WF}\text{)}\text{ =}\frac{\text{C}\text{×}\text{V}\text{T}}{\text{1000}\text{×}\text{V}\text{S}\text{×}\text{WF}}$$

Where:​​

C: Protein content obtained from the standard curve (μg)

V_T_: Total volume of the extraction solution (mL)

V_S_: Sample volume used for measurement (mL)

WF: Fresh weight of the sample (g)

1000: Unit conversion factor (μg → mg)
